# S2k-Guideline hand antisepsis and hand hygiene

**DOI:** 10.3205/dgkh000497

**Published:** 2024-09-06

**Authors:** Axel Kramer, Julia Seifert, Marianne Abele-Horn, Mardjan Arvand, Paul Biever, Alexander Blacky, Michael Buerke, Sandra Ciesek, Iris Chaberny, Maria Deja, Steffen Engelhart, Dieter Eschberger, Bernd Gruber, Achim Hedtmann, Julia Heider, Udo B. Hoyme, Christian Jäkel, Peter Kalbe, Horst Luckhaupt, Alexander Novotny, Cihan Papan, Hansjürgen Piechota, Frank-Albert Pitten, Veronika Reinecke, Dieter Schilling, Walter Schulz-Schaeffer, Ulrich Sunderdiek

**Affiliations:** 1 Institute of Hygiene and Environmental Medicine, University Medicine Greifswald, Greifswald, Germany; 2 German Trauma Society, Berlin, Germany; 3 Paul-Ehrlich-Society for Infection Therapy, Munich, Germany; 4 Robert Koch Institute, Department Infectious Diseases, Unit Hospital Hygiene, Infection Prevention and Control, Berlin, Germany; 5 German Society for Internal Intensive Care and Emergency Medicine, Berlin, Germany; 6 Austrian Society for Hospital Hygiene, Vienna, Austria; 7 German Cardiac Society, Düsseldorf, Germany; 8 Society of Virology, Aschaffenburg, Germany; 9 German Society for Hygiene and Microbiology, Münster, Germany; 10 German Society of Anaesthesiology and Intensive Care Medicine, München, Germany; 11 Society of Hygiene, Environmental and Public Health Sciences, Freiburg, Germany; 12 Vienna Regional Office of the Austrian Workers' Compensation Insurance, Vienna, Austria; 13 German Nursing Council, Berlin, Germany; 14 Professional Association of Orthopaedic and Trauma Specialists (BVOU), German Society for Orthopaedics and Trauma, Berlin, Germany; 15 German Society for Oral, Maxillofacial and Facial Surgery, Hofheim am Taunus, Germany; 16 Working Group for Infections and Infectious Immunology in the German Society for Gynecology and Obstetrics, Freiburg, Germany; 17 Dr. Jäkel, Medical Law, Pharmaceuticals Law, Medical Devices Law, Luebben, Germany; 18 Professional Association of German Surgery, Berlin, Germany; 19 German Society of Oto-Rhino-Laryngology, Head and Neck Surgery, Bonn, Germany; 20 German Society for Surgery, Berlin, Germany; 21 German Society for Pediatric Infectious Diseases, Berlin, Germany; 22 German Society for Urology, Düsseldorf, Germany; 23 German Society for Hospital Hygiene, Berlin, Germany; 24 German-speaking Interest Group of Experts for Infection Prevention and Consultants for Hospital Hygiene, Zurich, Switzerland; 25 German Society for Digestive and Metabolic Diseases, Berlin, Germany; 26 Department of Neuropathology, Medical Faculty of the Saarland University, Homburg/Saar, Germany; 27 German X-ray Society and German Society for Interventional Radiology and Minimally Invasive Therapy, Berlin. Germany

**Keywords:** hand antisepsis, hand disinfection, hygienic hand antisepsis, hygienic hand disinfection, surgical hand antisepsis, surgical hand disinfection, hand wash, alcohol-based hand rub requirements, wash lotion requirements, skin protection, skin care, adherence hand antisepsis, adherence hand disinfection, listing alcohol-based hand rubs, liability

## Abstract

The consensus-based guideline “hand antisepsis and hand hygiene” for Germany has the following sections: Prevention of nosocomial infections by hygienic hand antisepsis, prevention of surgical site infections by surgical hand antisepsis, infection prevention in the community by hand antisepsis in epidemic or pandemic situations, hand washing, selection of alcohol-based hand rubs and wash lotions, medical gloves and protective gloves, preconditions for hand hygiene, skin protection and skin care, quality assurance of the implementation of hand hygiene measures and legal aspects.

The guideline was developed by the German Society for Hospital Hygiene in cooperation with 22 professional societies, 2 professional organizations, the German Care Council, the Federal Working Group for Self-Help of People with Disabilities and Chronic Illness and their Family Members, the General Accident Insurance Institution Austria and the German-speaking Interest Group of Infection Prevention Experts and Hospital Hygiene Consultants.

## Abstract (de)

Die Konsensus basierte Leitlinie ist in folgende Themen untergliedert: Prävention nosokomialer Infektionen durch hygienische Händeantiseptik, Prävention postoperativer Wundinfektionen durch chirurgische Händeantiseptik, Infektionsprävention in der Bevölkerung durch Händeantiseptik bei epidemischer bzw. pandemischer Situation, Händewaschung aus sozialer und infektionspräventiver Indikation, Auswahl von Alkohol-basierten Händeantiseptika und von Waschlotionen, medizinische Handschuhe und Schutzhandschuhe, Voraussetzungen für die Händehygiene, Hautschutz und Hautpflege, Qualitätssicherung der Umsetzung der Maßnahmen der Händehygiene und rechtliche Aspekte.

Die Leitlinie wurde von der Deutschen Gesellschaft für Krankenhaushygiene in Zusammenarbeit mit 22 Fachgesellschaften, 2 Berufsverbänden, dem Deutschen Pflegerat, der Bundesarbeitsgemeinschaft Selbsthilfe von Menschen mit Behinderung und chronischer Erkrankung und ihren Angehörigen, der Allgemeinen Unfallversicherungsanstalt Österreich und der Deutschprachigen Interessengruppe der Fachexperten/innen für Infektionsprävention und Berater für Spitalhygiene erarbeitet.

## Introduction

In the guideline, the term “hand antisepsis” is used consistently instead of “hand disinfection”, which is commonly used in Germany, France, Austria and other European countries. The main reason is that antisepsis (Greek: anti=against, sepsis=putrefaction) refers to locally applied measures on or in living tissue to kill or reduce microorganisms or inactivate viruses. In the U.S., the criterium of episomatic application is the basis for the differentiation between antisepsis and disinfection. According to various U.S. regulations, antisepsis and disinfection are differentiated: the use of antimicrobial preparations for the reduction of microorganisms on the body surface is antisepsis. Therefore, antiseptics are covered by the same regulations as drugs and cosmetics (Federal Food, Drug, and Cosmetic Act). On the other hand, disinfection includes the killing or removal of pathogens on non-living materials. As opposed to disinfection, antimicrobial treatment of the body surface always has an antiseptic, but never a disinfecting effect.

The aim of prophylactic antisepsis is to prevent infection by decontaminating contaminated or colonized skin, mucous membranes, eyes and wounds, and if necessary also by local application in body cavities, to prevent the transfer of pathogens from contaminated or colonized areas to non-colonized areas of the body, interrupt the transfer of microbes and viruses to patients or healthy persons, protect the operating field from colonized areas of the body (i.e., skin, mucous membranes, wounds), to kill potential pathogens after accidental contamination or normalize dysbiosis [1], [2], [3]. 

Scope and purpose, methods, participating authors and societies are listed in the Guideline Report (Attachment 1 ). It should also be noted that this guideline was first published in German [4].

## Recommendations and justification

### 1. Prevention of nosocomial infections by hygienic hand antisepsis

The classifications of the recommendations for the following recommendation degrees as well as the consensus classification for the strength of consensus can be found in the Guideline Report (Attachment 1).

**1. Recommendation**

*Immediately before any possibility of transmission of infectious agents to the patient by the hands of nursing staff, doctors and those involved in the care process, hygienic hand antisepsis should be carried out.*

*As basis for action, the 5 moments of hand antisepsis introduced by the WHO in the clinical setting and the 4 moments in the outpatient setting should be observed by all staff involved in patient care:*

- *In the direct patient environment, hand rub before patient contact, before aseptic activities and after contact with potentially infectious material, *
- *in the extended patient environment, hand rub after each patient contact and after each contact with the immediate patient environment.*

*This applies regardless of whether non-sterile or sterile disposable medical gloves are put on after hand rub.*

**Recommendation degree:** ↑↑ 

**Strength of consensus: **>95%

Hygienic hand antisepsis is considered the most effective single measure worldwide for interrupting the transmission of infections in inpatient and outpatient health care facilities, in the outpatient care of people in need of nursing care, and in the nursing care of nursing-home residents, to prevent health care-associated as well as community-associated infections [5], [6], [7], [8], [9], [10], [11], [12], [13], [14]. The first publication by Ignaz Philipp Semmelweis over 150 years ago already impressively demonstrated the effectiveness of hand antisepsis [15], [16]. Likewise, hand antisepsis controls the spread of multi-resistant pathogens (MDRO) and reduces the incidence of associated colonizations and infections [17], [18], [19], [20], [21], [22], [23], [24]. Finally, the effectiveness of hand antisepsis in interrupting bacterial and viral nosocomial outbreaks has been demonstrated [25], [26], [27], [28], [29]. Furthermore, hand antisepsis contributes to self-protection [30].

The hands of staff are contaminated with pathogens and are the most important carriers of pathogens [5], [31], [32], [33], [34], [35], [36], [37]. Therefore, hygienic hand antisepsis must be carried out during measures on patients and after contact with contaminated surfaces. The WHO has summarized the indications for hand antisepsis in five indication groups (the “five moments”) as the basis for education and training on hand antisepsis. At the same time, this should facilitate the recognition of indications in the workflow and the adherence to them [10].

Hygienic hand antisepsis reduces pathogens on the hands (transient flora) to such an extent that their further spread is prevented. Hygienic hand antisepsis leads to a significantly higher reduction in the number of pathogens than hand washing and thus offers greater safety for infection control [38], [39], [40], [41], [42], [43], [44], [45], [46], [47], [48], [49], [50], [51], [52], [53], [54], [55], [56], [57], [58], [59], [60], [61], [62]. In addition, the skin is less affected than by soap washing [35], [63], [64], [65], [66] and the environmental impact is also lower [67].

**2. Recommendation**

*After removing disposable medical gloves (non-sterile or sterile gloves), hand antisepsis should be performed.*

**Recommendation degree:** ↑↑ 

**Strength of consensus:** >95%

Hand antisepsis is required after removing pathogen-free disposable medical gloves because gloves can become perforated and go unnoticed during wear [68], [69], [70], [71], contaminating the hand [72]. At the same time, hands can become contaminated if gloves are not removed properly. Hand antisepsis prevents the further spread of pathogens.

An observational study revealed deficits in this regard. Only 18.6% of the nursing staff performed hand antisepsis before putting on gloves, 65% after taking them off and 47.2% when changing gloves [73]. If the glove is removed correctly, the finger of the hand from which the glove was first removed goes into the glove at the wrist of the other hand and then pulls it down without touching it from the outside. This procedure is often not used due to ignorance. Instead, the gloves are removed by grasping the upper cuff at the wrist with the thumb and forefinger and pulling down, which makes touching the outer surface unavoidable. However, the outer surface may be contaminated by contact with infectious material.

**3. Recommendation**

*In case of visible contamination, hand washing may be considered after removing surgical gloves.*

**Recommendation degree:** ↔ 

**Strength of consensus: **>95%

Due to the accumulation of sweat in the glove (so-called glove juice), hand washing can be performed if individually appropriate. At the end of the surgical program, the use of a skin-care lotion is recommended [74]. 

**4. Recommendation**

*Hand antisepsis should be performed before food prep* *a* *ra* *tion and distribution.*

**Recommendation degree:** ↑↑

**Strength of consensus: **>95%

As hand antisepsis with ABHR is significantly more tolerable than soap washing [75], [76], hand antisepsis, depending on the frequency of hand washing, can help to reduce skin exposure through repeated hand washing. In cases of infectious rhinitis, e.g., caused by influenza, parainfluenza, rhino, boca and RS viruses, or bacterial pathogens such as *Streptococcus pneumoniae* and *Haemophilus influenzae*, hand antisepsis reduces the pathogen load on the hands more than hand washing [35], [39], [53].

**5. Recommendation**

*Patients should be informed about the importance of hand antisepsis when entering and leaving the patient’s room, before eating, after going to the toilet and before contact with wounds and catheters.*

**Recommendation degree:** ↑

**Strength of consensus:** >95%

Patient-performed hand antisepsis is of particular importance for patient protection, because its infection prevention benefits have been demonstrated in various populations even outside health care facilities [30], [77], [78], [79], [80], [81], [82]. 

The importance of the involvement of patients in infection control is obvious, as patients are potential recipients and transmitters of nosocomial infections [56], [83], [84], [85]. If they are given basic knowledge about how they can contribute to their own protection, they will not only behave accordingly, they will also feel safer, which can support the healing process. Patients' participation in the hospital's hand hygiene program has significantly improved their hand hygiene practices before eating and tended to improve them after using the toilet [84]. For didactic reasons, the assignment to the 5 WHO moments is also recommended for patients [83], [84]. First experiences confirm this approach [84], [85]. 

An additional effect of involving patients and their family members was that the choice of the hospital was positively influenced [86].

**6. Recommendation**

*For hand antisepsis, the hand rub should be applied on the dry (!) hand without adding water, depending on the situation, before or during a work-step or afterwards.*

**Recommendation degree: **↑↑

**Strength of consensus: **>95%

The hand rub must be applied to the dry (!) hand, because alcohol-based agents lose their effectiveness when diluted with water [87].

**7. Recommendation**

*A rubbing technique should be chosen which ensures that both hands are completely moistened for the ex* *po* *sure* *time declared by the manufacturer. To ensure sufficient wetting, at least 3 ml of ABHR should be applied. The solution should be distributed evenly by rubbing the hands together so that the entire surface of the hand, i.e. fingertips, nail folds, thumbs, spaces between fingers, inner and outer surfaces as well as wrists are completely moistened. Nail folds and fingertips should be treated particularly intensively.*

**Recommendation degree:** ↑↑

**Strength of consensus: **>95%

Complete wetting of the hands is necessary because the hand antiseptic cannot develop its effect in wetting gaps. If the user is not aware of the risk of incomplete wetting and has not made it visible using fluorescence during hand antisepsis training, wetting gaps result [88].

The antiseptic should be rubbed particularly on the fingertips, nail folds and thumbs [89], [90], which is often ignored [91]. When comparing the sequence of movements specified in DIN EN 1500 [92] with self-selected rubbing techniques, with the focus on wetting the above-mentioned parts of the hand, no advantage could be demonstrated for the sequence of movements in the test standard [89], so that the rubbing model recommended in DIN with 6 individual steps does not have to be adhered to. However, an individually practiced rubbing technique is recommended to ensure that the implementation is as standardized as possible.

If <2 ml of hand antiseptic is applied, the wetted area is significantly reduced [93]. To ensure adequate wetting of the hands, a minimum volume of 3 ml must be used [89], [93]. The dispensed quantity from the ABHR dispenser must be calibrated accordingly.

**8. Recommendation**

*The surface of the hand should remain moist for the duration of the exposure time declared by the manufacturer. The minimum exposure time should be 15 s. After the exposure time has elapsed, the hands should not be dried.*

**Recommendation degree:** ↑↑

**Strength of consensus:** >95%

The exposure time declared by the manufacturer is based on testing the effectiveness of the hygienic hand antisepsis in accordance with DIN EN 1500 [92] and is the basis for inclusion in the disinfectant list of the Association for Applied Hygiene [94].

In 2020, the German “Clean Hands” campaign suggested shortening the rubbing time to 15 s [95] because it was proven that by training in hand antisepsis, a similar degree of hand wetting can be achieved in 15 s and 30 s [96]. In accordance with this, the effectiveness did not differ between the 15 s and 30 s application time in volunteers [97]. In the daily ward routine in a neonatological and gynecological ward, a comparison of 15 s vs. 30 s hand rubbing showed that the same antiseptic efficacy was achieved on the hands. However, by shortening it to 15 s, adherence to hand antisepsis increased significantly or tended to increase [98], [99].

When determining the number of indications for hand antisepsis, the sum of 3 shifts in a surgical intensive care unit (ICU) resulted in 271, 188 and 182 indications [100], in an internal medicine ICU 271, 163 or 134 indications [100], in a neurological ICU 124 indications [100], and in a gynecological ward, just in the day shift, an average of 138 indications for hand antisepsis was recorded [99]. The reason for increased adherence when the exposure time is shortened to 15 s is likely to be that, given the large number of indications, the willingness to carry out hand antisepsis increases due to the time saved. Observations showed that hand antisepsis was carried out by doctors for an average of only 8.5 s and by nursing staff for only 6.6 s [101], [102]. This underlines the usefulness of shortening it to 15 s.

After the exposure time has elapsed, the hands are not dried in order to not interrupt the after-effect and not mechanically remove lipids emulsified from the skin.

**9. Recommendation**

*Visible contamination on the hands should be removed. The hands should then be dried, and hand antisepsis should be performed.*

**Recommendation degree:** ↑

**Strength of consensus:** >95%

Visible contamination of the hands, e.g., with blood, secretions or excretions, must be removed before hand antisepsis; otherwise, the effectiveness of the antisepsis is not guaranteed [87], [103]. Before the subsequent hand antisepsis, the skin must be dried to avoid a dilution effect of the ABHR.

**10. Recommendation**

*Before introducing a new ABHR in a team, the acceptance of the change should be evaluated.*

**Recommendation degree:** ↑

**Strength of consensus:** >95%

Since different alcohols are used alone or in combination (ethanol, propan-1-ol, propan-2-ol), acceptance should be determined through a trial introduction before switching, especially since, in contrast to ethanol, 60% propan-1-ol can have an irritating effect on both healthy and atopic skin [104]. This particularly affects subjective acceptance, which is influenced, for example, by skin care additives [105].

Instead of the predominantly used hand rubs in solution form, alcohol-containing gels or foams are also available for hygienic hand antisepsis. The increased viscosity is sometimes perceived as more pleasant than solutions. A gel makes it difficult for the product to drip onto the floor. On the other hand, residue remains on the hands, which can make the skin feel sticky [106], [107]. Therefore, acceptance by employees should be evaluated, especially before the introduction of such products [106], [107], to rule out a negative influence on compliance. It should be noted that when using gels and foams, hands remain wet for significantly longer than 30 s, especially with products with a low alcohol content. It is unclear whether foaming agents have an adverse effect on skin tolerance. The use of the foam stabilizer PEG-12 is only considered safe on intact skin, not on damaged or irritated skin. Contact allergies to PEG 400 are possible in approximately 4% of cases in patients with damaged skin [87]. Alcohol absorption could also be increased, which could be toxicologically relevant for propanols [108]. 

**11. Recommendation**

*If gels or foams containing alcohol are used instead of liquid hand rubs, the efficacy should be proven according to the national and European test standards for the respective application form.*

**Recommendation degree:** ↑↑

**Strength** **of** **consensus:** >95%

A prerequisite for the use of gels or foams for hand antiseptics is the declared proof of efficacy for the given form of application [92], [103], [109].

### 2. Prevention of surgical site infections by surgical hand antisepsis

**12. Recommendation**

*Surgical hand antisepsis should be performed by all staff involved in the aseptic area of the surgery, as well as before other interventions with the same requirements for asepsis as in surgery.*

**Recommendation degree:** ↑↑

**Strength of consensus:** >95%

Surgical hand antisepsis is to be performed by all staff involved in the aseptic area of surgery (surgeons, OR assistants, instrument nurses). It should also be performed before other interventions that have the same level of asepsis requirements as a surgical intervention. This is to reduce the pathogen load as far as possible, especially of the resident skin flora. 

Only indirect evidence can be derived for surgical hand antisepsis, because a study design with reduced adherence is not ethically justifiable. The indirect evidence stems from the significantly higher SSI rate with perforated compared to non-perforated surgical gloves [110], [111]. It has been experimentally demonstrated that between 10^3^ and 10^4^ colony-forming units (cfu) can reach the surgical wound through glove lesions from bare hands without previous antisepsis [111], [112]. In contrast, the amount transmitted from hands after previous antisepsis was <100 cfu [113]. Consequently, in the event of intraoperative damage of surgical gloves, the number of microorganisms that enter the surgical wound with the sweat retained by the glove (glove juice) is kept low by the preceding surgical hand antisepsis, thus reducing the risk of SSI [112], [113]. It should be noted that according to DIN EN 455-1 [114], defects may already be present in 3 out of 80 or 4 out of 120 tested gloves; the “acceptable quality level” (AQL) is ≤1.5 in the case of brand-new, unused sterile surgical gloves. Surgical gloves are perforated in up to 40% of procedures, which is either noticed or unnoticed [68]. In surgical gloves perforated during the wearing process, bacterial transfer through the perforation was detectable after a wearing time of 90 min [69], [70], [71]. Based on the test model for surgical hand antisepsis, it can be assumed that its effect lasts for about 3 h. However, no studies have shown that surgical hand antisepsisis is required again after this time.

The following incident underlines the importance of surgical hand antisepsis: the use of a non-medicated soap instead of an iodine-containing hand rub caused an outbreak of SSI [115]. 

**13. Recommendation**

*The operating unit should be entered with clean hands. For washing, hands and forearms (up to the elbows) should be washed for about 30 s with a hand-washing product. Fingertips should point upwards and the elbow downwards. Afterwards, hands should be dried with a low-germ textile or paper towel.*

**Recommendation degree:** ↑↑

**Strength of consensus:** >95%

A study has shown that surgical workers’ hands were contaminated with up to 1 lg of bacterial spores, particularly *Clostridioides* spores, upon entering the healthcare facility [116]. When surgical hand antiseptics were tested, large numbers of spore-forming bacteria (predominantly *Clostridioides* spp.) were detectable in the glove juice of the subjects [116]. The findings emphasize the necessity of thorough hand washing once, at the latest in the operating room airlock, before surgical hand antisepsis [117], since alcohol-based hand antiseptics are not effective against bacterial spores [87]. 

If no washing area is available in the operating room airlock, an easily accessible hand-washing basin should be provided as an alternative. 

Hand washing for longer than 30 s should be rejected because of potential skin damage, especially since this does not achieve any further reduction of resident skin flora [118], [119], [120].

Final hand drying serves to remove residual pollution, including skin flora [121], and to ensure dry hands so that the efficacy of the ABHR is not compromised by dilution effects [87]. 

**14. Recommendation**

*Prior to consecutive surgeries, a repeat preoperative hand wash may be omitted, unless the hands are visibly soiled, or if there is no anticipated interim contamination with bacterial spores.*

**Recommendation** **degree:** ↔

**Strength of consensus:** >95%

The rationale for not performing a repeat hand washing if the hands are clean is that the surgical hand antisepsis is effective without the need for additional washing with soap [116], [122], [123], [124], [125], and each handwashing carries an additional risk of irritation [75]. 

**15. Recommendation**

*If activities that pose a risk of contamination with bacterial spores were performed between surgeries, a repeat handwashing should be conducted before resuming surgical procedures.*

**Recommendation degree:** ↑

**Strength of consensus:** >95%

If activities that pose a risk of contamination with bacterial spores are performed between surgeries, a repeat hand washing should be conducted before resuming surgical procedures. In a study involving 71 healthcare workers, the average spore contamination of hands after 9 hours of work was 468 cfu/hand, with *Bacillus subtilis* and *Bacillus cereus* being the predominant organisms. *C. difficile* was detected only in one case [126]. The risk of spore contamination of hands exists, for example, after contact with dust, care of patients with *C. difficile*-associated diarrhea, colonoscopy, and after using the toilet.

**16. Recommendation**

*Hands and forearms should not be treated with a nail brush for cleaning or antisepsis. If fingernails are soiled, they should be cleaned with a soft, thermally disinfected (or sterile) plastic brush, and if necessary, with a wooden stick or metal nail cleaner.*

**Recommendation** **degree:** ↑↑

**Strength of consensus:** >95%

The use of a brush irritates the skin without achieving any additional effect on the efficacy of preoperative antisepsis of hands and forearms [75], [127], [128]. On the contrary, brushing releases bacteria of the resident skin flora in significant amounts (p<0.001) [128], [129]. In case of dirty fingernails, this pathogen reservoir, which contains most of the hand flora [130], can only be eliminated mechanically [126].

**17. Recommendation**

*There should be an interval of >10 minutes between hand washing and surgical hand antisepsis.*

**Recommendation degree:** ↑↑

**Strength of consensus:** >95%

A shorter interval can tend to or significantly reduce the effectiveness of ABHR due to the dilution effect of residual humidity present in the epidermis [116], [117], [122], [123]. Additionally, the increase in the number of pathogens on the hands associated with soap washing, with [129], [131], or without the use of a brush [116], [124], [132], may also reduce the efficacy [133]. In emergency situations, it may not be possible to adhere to the recommended interval.

**18. Recommendation**

*Before putting on operating-area clothing, hygienic hand antisepsis must be performed and should be repeated any time when entering the OP theatre.*

**Recommendation degree:** ↑↑

**Strength of consensus:** >95%

Hand antisepsis reduces the contamination of operating-area clothing and therefore minimizes the introduction of pathogens into the OP theatre.

**19. Recommendation**

*Surgical hand antisepsis means that medical staff completely wet their hands and forearms with an ABHR. Exposure time is dependent on the manufacturer’s recommendationss. Gaps of wetting must be avoided; attention should be paid to fingertips, nailfolds and spaces between fingers.*

**Recommendation degree:** ↑↑

**Strength of consensus:** >95%

A practiced, individually standardized rub-in technique ensures uniform wetting of the hands better than if the distribution of the hand rub is performed differently each time (76). During surgical hand antisepsis, first the skin areas of the hand, then the forearm up to the elbow and subsequently the hands are wetted again. In the hand antisepsis phase, the main focus should be on fingertips, nail folds and interdigital spaces when rubbing in the solution, and complete wetting should be achieved. To avoid recontamination, care must be taken to ensure that not treated with antiseptic areas of skin are touched when performing hand antisepsis [134]. Before donning gloves, the hands should be completely dry. 

The exposure time declared by the manufacturer is based on test results from German and European test specifications [135], [136]. It contains the minimum exposure time required for the specific hand antiseptic, which must have reached the efficacy of the reference standard propan-1-ol 60% v/v tested in parallel for a duration of 3 min. Depending on the ABHR, the exposure time varies between 1 and 5 min [94]. It must be considered that the standard test method [135] only covers wetting of the hands and therefore only the effect on the hands is determined. However, since surgical hand antisepsis in practice also involves the forearms, a surgical hand antiseptic with a declared exposure time of 1.5 min was examined to determine whether the effect is achieved both on the hands and the forearms. As a result, the following procedure proved to be effective. First, both hands were wetted (10 s), and in the 2^nd^ step both forearms were also (10 s). This was followed by the hand antisepsis phase (70 s) by rubbing the hands. The number of portions applied had no influence on efficacy, as long as the hands were kept wet with the ABHR for the duration of the exposure time [137].

**20. Recommendation**

*Hands should be air dried after surgical hand antisepsis before gloving*.

**Recommendation** **degree:** ↑↑

**Strength of consensus:** >95%

Hands should be dry before donning gloves to minimize the risk of perforation [138] as well as skin irritation [116], and to optimize the efficacy of ABHR. Furthermore, gloving wet hands is difficult.

### 3. Prevention of infections by hand antisepsis in the community during epidemic or pandemic situations

During the COVID-19 pandemic, the areas in which hand antisepsis was performed multiplied, by placing dispensers for ABHR in the entrance areas of health care facilities, public buildings, supermarkets, stores, and the like. Since SARS-CoV-2 could be recultivated from hands for up to 9 h after experimental contamination [139], this preventive measure is logical, although it is currently unclear to what extent the extension of hand antisepsis to public facilities has had an impact on preventing the spread of SARS-CoV-2. Thirteen of 16 studies on the effectiveness of implementing hand antisepsis in community settings reported a protective effect against viral influenza, SARS, and COVID-19 (p<0.05); however, the studies were of limited methodological quality [140]. Future studies are needed to determine the circumstances under which and how frequently ABHR should be used in the general population to develop specific behaviors in the community, as well as in specific communities, such as schools and nursing homes, during epidemics or pandemics [140]. A previous analysis [141] found moderate to poor evidence of reductions in both viral influenza and other respiratory infections from hand hygiene interventions in schools when low- to middle-income settings were present. In contrast, there was high-quality evidence of small reductions in respiratory infections in child-care facilities and large reductions in respiratory infections in poor communities not connected to electricity and water. Furthermore, moderate- to high-quality evidence exists showing no impact on secondary transmission of influenza in households where an index case had already occurred. The rationale for extending hand antisepsis to the general population is indirectly supported by the fact that in a prospective, controlled, randomized trial in a public administration setting, additional provision of hand antiseptic significantly reduced respiratory infections and diarrhea, including resulting days of absence, over the course of one year compared with controls performing only social hand washing [30]. Intervention in the form of hand hygiene also reduced the incidence of respiratory infections and diarrhea in kindergartens and schools, albeit to varying degrees [142], [143].

In conclusion, although hand antisepsis has the potential to reduce transmission of influenza, other acute respiratory infections, and gastrointestinal infections, efficacy varies depending on the setting and personal discipline in terms of compliance. Because ABHR is not associated with any health hazard [144], hand antisepsis with ABHR is recommended in public areas in epidemic or pandemic situations as a supportive protective measure.

### 4. Hand washing for social and infection prevention indication

**21. Recommendation**

*Hand washing should be performed only when hands are dirty or after using the rest room. Subungual spaces, nail folds and heavily soiled hands should be cleaned already at home.*

**Recommendation degree:** ↑↑

**Strength of consensus:** >95%

Hand washing after soiling and for aesthetic reasons fulfills a social and general hygiene requirement, but has no influence on the rate of nosocomial infections due to the lack of microbicidal effect of soaps under hospital conditions [35].

Dirty hands, subungual areas and nail folds should be cleaned already at home. This allows the skin lipids emulsified and rinsed off by hand washing to be restituted in the time period leading up to entry into the healthcare facility, reducing the exposure of the skin to subsequent hand washing or hand antisepsis. Hand washing should be limited to the specified indications, since hand washing, in contrast to alcohol-based hand antisepsis, is associated with the risk of skin irritation. Frequent hand washing causes the stratum corneum to swell; alteration and emulsification of intercellular lipid bilayers can occur, and the lipids are washed away along with water-soluble moisturizing factors and antimicrobial protective factors. As a result, the skin dries out, the stratum corneum can break open, and inflammation occurs in the epidermis and cutis with keratinization disorders, which can result in an irritant dermatosis that may be difficult to treat [75]. If hands subjected to occupational stress are washed four times within 1 hour, the time period for the skin parameters to normalize is insufficient [127]. Although skin lipids are also emulsified in the stratum corneum by ABHR and forced out of their structural arrangement, they remain substantially on the skin – unless rinsed off [66]. The better skin tolerance of ABHR compared to soaps has been proven by a large number of experimental findings and application studies [76], [145].

**22. Recommendation**

*If there is a risk of transmission of bacterial spores, protozoa, oocysts or helminths, thorough hand washing should be performed after unprotected hand contamination or after removing the disposable medical gloves worn for protection and subsequent hand antisepsis.*

**Recommendation degree:** ↑↑

**Strength of consensus:** >95%

With alcohols, even in combination with peracetic acid, sporicidal activity cannot be achieved with tolerable skin compatibility or practical exposure time [146]. Furthermore, protozoa, oocysts and helminths are not killed by ABHR [87]. Therefore, if there is a risk of hand-borne transmission of bacterial spores, protozoa, oocysts or helminths, due to the surface activity of soaps and the mechanical removal that can be achieved by rinsing [147], [148], thorough hand washing with soap and water is recommended after removing disposable medical gloves [149], [150]. If there is no hand-washing facility in the patient’s room or the associated sanitary cell, the nearest washing facility must be used, avoiding contamination of the surroundings, e.g., door handle or railing, on the way there. Before washing hands, the hands are to be treated antiseptically with ABHR so that bacteria, particularly Gram-negative ones, that may adhere to the hands do not enter the siphon. Biofilm formation occurs in the siphon and pathogens are emitted into the environment when water is subsequently introduced [151], [152], [153].

For laboratory work with spores in the workbench, preparations containing peracetic acid can be used for a short time.

An aqueous formulation based on hydrogen peroxide and nitrite has sporicidal activity [154]. Since highly reactive nitrogen compounds such as peroxynitrite are formed, which have mutagenic effect and might be carcinogenic [155], [156], they cannot be used for hand antisepsis.

**23. Recommendation**

*After each* *washing of hands, any soap residue should be thoroughly rinsed away and hands should be carefully dried with a disposable towel (textile or paper).*

**Recommendation degree:** ↑

**Strength of consensus**: >95%

Rinsing off soap residues and subsequent hand drying serve to remove residual dirt, including skin flora released to the surface [121]; drying ensures that, in the event of subsequent hand antisepsis, the efficacy of the alcohol-based hand rubs is not impaired by dilution effects [87].

### 5. Selection of alcohol-based hand rubs and wash lotions

**24. Recommendation**

*For both hygienic and surgical hand antisepsis, ABHR without added antiseptic agents should be used.*

**Recommendation degree:** ↑↑

**Strength of consensus:** >95%

Although the efficacy of alcohols for surgical hand antisepsis can also be achieved with antiseptic soaps based on chlorhexidine digluconate or with aqueous PVP-iodine solution if the exposure time is appropriate [157], antiseptic soaps should be rejected for hand antisepsis because of poor skin tolerance and PVP-iodine because of thyroid gland hazard. In a recent crossover study, the reference alcohol propan-1-ol 60% was equivalent to antiseptic soaps in the immediate value of lg reduction, but after 3 h, propanol was significantly superior [158]. For soaps containing chlorhexidine digluconate, another reason for rejection for use in hand antisepsis is the risk of resistance development with cross-resistance to antibiotics [159]. Because of the poorer efficacy of antiseptic soaps, alcohol-based preparations have become established in the majority of cases worldwide [160].

Aqueous-based iodophores pose a hazard due to the dermal resorption of released iodine through the intact skin. Depending on the duration of application, iodine resorption can reach critical iodine concentrations for the hyperthyroid and possibly also for the euthyroid thyroid. Another disadvantage of aqueous-based iodophores is the required exposure time of 60 s for hygienic or 5 min for surgical hand antisepsis [94]. When using iodine-based hand rubs, the following contraindications must be observed even with a single application: hypersensitivity to iodine, hyperthyroidism, autonomous thyroid adenoma, and radioiodine therapy. In case of pregnancy, anamnestically known thyroid diseases and the presence of nodular goiter, the application is only justifiable if the thyroid function is monitored. In case of long-term use, monitoring of thyroid function is recommended even in patients with a history of a healthy thyroid. Due to the risk to the thyroid gland, especially in the case of nutritional iodine deficiency, use over a period of months or years should not be regarded as risk-free, even in healthy individuals. For predisposed thyroid glands with autonomous tissue regions (i.e., regions which cannot be stimulated by exogenous thyroid-stimulating hormone) exceeding a critical volume, there is a risk of triggering hyperthyroid metabolic derailments even with relatively small amounts of iodine [161], [162], [163], [164], [165], [166], [167], [168], [169], [170], [171], [172], [173], [174], [175], [176], [177], [178], [179]. Thus, iodophores, in both aqueous and alcoholic bases, are not a product of choice for hand antisepsis. 

Aqueous solutions based on chlorine releasers or peroxides as well as liquid washing preparations with added antiseptics are also no alternative to the use of ABHR due to their lower efficacy and poorer skin compatibility compared with ABHR, their poorer spreading behavior on the skin and their longer evaporation time [35], [75], [180], [181], [182], [183], [184]. In contrast, the alcohols ethanol and propan-2-ol used for hand antisepsis do not cause any change in skin barrier properties and do not have an increased irritation potency even on preirritated skin [87]. On the contrary, ethanol-based hand antisepsis (EBHR) led to an improvement in skin condition in nurses who previously used only antiseptic soaps instead of ABHR [182], [184]. In contrast, propan-1-ol can apparently have an irritating effect on the skin, as shown in a recent systematic review [104]. Neither ethanol nor both propanols have a sensitizing effect [87].

For the removal of pathogens that have reached the hands (transient skin flora), and likewise for the preoperative removal of resident skin flora by ABHR, a longer-lasting (so-called remanent or remaining) effect of the hand rub by antiseptic additives is not required, because there is no evidence that greater efficacy of both hygienic and surgical hand antisepsis is achieved by remanent-acting additives to ABHRs [185]. On the other hand, remanent-acting additives such as chlorhexidine digluconate, octenidine dihydrochloride, polihexanide, quaternary ammonium compounds (e.g., benzalkonium chloride), as well as phenol derivatives and triclosan, when added to alcohols, are associated with the risks of reduced skin tolerance, sensitization or resorptive side effects [104], [185], [186], [187], [188], [189], [190], [191], [192], [193], [194], [195], [196], [197], [198], [199], [200], [201], [202], [203], [204], [205], [206]. In rare cases, chlorhexidine may cause anaphylactic reactions as an immediate allergic reaction after a single application [203], [204]. With long-term use of chlorhexidine, triclosan, and benzalkonium chloride, resistance development is possible, in some cases with cross-resistance to antibiotics [159], [207], [208], [209], [210], [211], [212], [213], [214], [206]. In 2015, ECHA banned the use of triclosan in antiseptics for human use because of environmental hazards [215]. In the same year, the U.S. Federal Drug Association banned the use of triclosan in soaps [216].

**25. Recommendation**

*For hand antisepsis, alcohol-based hand rubs with added refatting agents and possibly other skin-soothing additives should be selected.*

**Recommendation degree:** ↑

**Strength of consensus: **>95%

The skin's tolerance is improved by the addition of refatting agents [65], [217], [218], [219]. The inclusion of glycerol also enhances the skin’s barrier function [220], [221]. When used in conjunction with proper skin care, the application of ABHRs is not associated with the risk of irritant dermatitis [65]. An ethanol-based hand antiseptic gel contains allantoin as an active ingredient, which has soothing properties [64], [222], [223], anti-inflammatory effects [224], and promotes wound healing [225]. Whether these effects occur in an alcohol-based antiseptic gel has not been studied.

**26. Recommendation**

*The selection of hand antiseptics for prophylactic purposes should be based on the disinfectant list of the Association for Applied Hygiene („Verbund für Angewandte Hygiene“; VAH).*

**Recommendation degree:** ↑

**Strength of consensus: **>95%

The disinfectant list of the Association for Applied Hygiene („Verbund für Angewandte Hygiene“; VAH) ensures the reliable fulfillment of efficacy requirements for the selection of hand antiseptics for the following reasons: It includes a compilation of all products that have a valid VAH certificate at the respective publication date. This certificate is issued when the efficacy requirements published by the Disinfectant Commission are met. The assessment process involves the evaluation of expert reports and test reports from two independent, accredited testing laboratories by impartial experts who are not affiliated with the manufacturers. The proof of efficacy for the specific intended use, as well as the stated concentrations and exposure times, are based on a minimum of two expert reports with their corresponding test reports. These evaluations rely on scientifically validated testing methods developed by VAH [94], the German Association for the Control of Virus Diseases („Deutsche Vereinigung zur Bekämpfung der Viruskrankheiten e.V.“; DVV) [226], [227], [228], or comply with relevant European standards.

**27. Recommendation**

*Hand antiseptic measures mandated by authorities should be based on the list of disinfectants tested and recognized by the Robert Koch Institute (RKI).*

**Recommendation degree: **↑↑

**Strength of consensus:** >95%

According to § 18(1) of the Infection Protection Act [229], only agents and methods that are included in the list of disinfectants and disinfection methods tested and recognized by the Robert Koch Institute (RKI) [230] may be used for antiseptic and/or disinfectant measures mandated by authorities. The underlying test methods differ, for example, in the type of test organisms used. For instance, Range of Action A generally includes mycobacteria in addition to vegetative bacteria and fungi, including fungal spores. Mycobacteria typically have higher requirements for antiseptics due to their chemotolerance.

**28. Recommendation**

*Given a risk of spreading of non-enveloped viruses with low lipophilicity (e.g., adeno-, noro-, rotaviruses), ABHR labeled as “limited virucidal Plus” should be used. In the case of more resistant non-enveloped hydrophilic viruses (e.g., hepatitis A-, hepatitis E-, coxsackie-, echo-, papillomaviruses), hand rubs labeled as “virucidal” should be used.*

**Recommendation degree:** ↑↑

**Strength of consensus:** >95%

The spectrum of action of ABHR includes bacteria, yeasts, and enveloped viruses [87]. To inactivate non-enveloped viruses, either a high ethanol concentration (>77% v/v) or synergistic combinations of ethanol with enhancing additives at lower ethanol concentrations are required [231], [232], [233], [234], [235], [236], [237], [238], [239], [240], [226]. This leads to the following implications for the selection of ABHR. For non-enveloped viruses with low lipophilicity (e.g., noro-, adeno-, rotaviruses), the hand rub must meet the criteria for labeling as “limited spectrum of virucidal activity Plus”. For non-enveloped viruses with high lipophilicity (e.g., hepatitis A-, hepatitis E-, coxsackie-, echo-, papillomaviruses), hand rubs labeled as “virucidal activity” should be used [83], [144], [226]. To qualify for the labeling “limited spectrum of virucidal activity”, “limited spectrum of virucidal activity Plus”, or “virucidal activity”, the requirements outlined in the DVV/RKI guideline or the European Norm [227], [241] must be met. This differentiation is highly relevant in practice, because non-enveloped viruses can be transmitted via hands when conventional hand rubs without the spectrum of action “limited virucidal Plus” or “virucidal” are used, leading to nosocomial infections and outbreaks [29], [144].

**29. Recommendation**

When there is a risk of hand contamination with mycobacteria, it is advisable to use hand rubs labeled as “tuberculocidal” or “mycobactericidal”, depending on the specific pathogen

**Recommendation degree: **↑

**Strength of consensus:** >95%

*M. tuberculosis* is only killed by alcohol-based formulations labeled as “tuberculocidal”, while atypical mycobacteria (e.g.,* M. chelonae* and *M. fortuitum*) are only eradi-cated by alcohol-based formulations labeled as “mycobactericidal”. The testing standard for this is DIN EN 14348 [242]. When caring for patients with extrapulmonary tuberculosis with risk of hand contamination, products labeled as having tuberculocidal efficacy for *M. tuberculosis* or mycobactericidal efficacy for other mycobacterial species should be used [242]. There is no evidence to suggest that hand transmission is possible with open pulmonary tuberculosis.

**30. Recommendation**

*Ethanol-based hand rubs may be considered preferable for hand antisepsis.*

**Recommendation degree: **↔

**Strength of consensus:** >95%

Unlike ethanol, 60% propan-1-ol can be irritating to both healthy and atopic skin [104]. Tissue compatibility studies on peritoneal explants have shown that 80% ethanol is better tolerated than 60% propan-2-ol [243]. This could be advantageous for use on irritated or particularly sensitive skin. Additionally, ethanol has significantly lower inhalation toxicity compared to the two propanols [244], [245], [246], although no intoxications from inhalation of alcohol released during hand antisepsis have been reported for any of these alcohols. Ethanol is absorbed only in trace amounts, well below any toxic, carcinogenic, mutagenic, or fetotoxic risk, and far below the ethanol intake from foods containing hidden, undeclared ethanol content, such as apple juice and kefir [144]. Thus, there is no systemic hazard associated with its use. Although the practice of routine hand rub on the day of application and the morning after in night-shift workers may result in urinary ethyl glucuronide levels exceeding the legally established threshold for assessing the ability to drive a motor vehicle in Germany [247], [248], it does not pose a health risk. 

Evidence of the reproductive toxicity of ethanol comes from the consumption of alcoholic beverages by pregnant individuals, meaning all knowledge about the reproductive toxicity of ethanol pertains to the abuse of alcoholic beverages. There is no epidemiological evidence of toxic risk to workers through the use of EBHR in healthcare facilities [144]. There is also no hint of toxic hazards, including carcinogenesis, associated with ABHR based on propan-1-ol and propan-2-ol. Due to differences in metabolically mediated physiological blood levels, the increase in blood alcohol levels over baseline after ABHR was approximately 157-fold for ethanol, over 1,800-fold for propan-1-ol, and over 10,000-fold for propan-2-ol [144]. As a result, the preference for EBHR can be derived based on their better physiological adaptation (occurrence and breakdown of ethanol in the body), especially for sensitive patients (e.g., newborns, infants, patients with respiratory diseases).

**31. Recommendation**

*Liquid hand wash lotions should be used for handwashing, and the use of solid soap bars should be prohibited.*

**Recommendation degree:** ↑↑

**Strength of consensus:** >95%

Solid soaps should not be used, as repeated contamination with both Gram-positive and Gram-negative bacteria has been demonstrated under usage conditions [249], [250], [251], [252]. After the introduction of a liquid soap instead of solid soap, the rate of nosocomial infections dropped from 4.2% to 2.2% over the course of a year, underscoring the risk assessment [252].

**32. Recommendation**

*Liquid hand wash lotions should be free of pathogens and microbiologically safe for use.*

**Recommendation degree:** ↑↑

**Strength of consensus:** >95%

Hand wash lotions fall under cosmetic regulations and have been required to meet the cosmetic standards since 2015 in terms of being free of pathogens and not exceeding allowable colony counts, which is ≤10^3^ cfu/ ml (for use on children <10^2^ cfu/ml) [253], [254].

**33. Recommendation**

*The pH level of hand wash lotions should preferably be slightly acidic (pH 5.5), but in any case, it should be not above neutral.*

**Recommendation degree:** ↑

**Strength of consensus:** >95%

Hand wash lotions with slightly acidic pH value that corresponds to the pH of the skin are better tolerated by the skin than alkaline soaps [255], [256].

### 6. Medical gloves and protective gloves

**34. Recommendation**

*Pathogen-free disposable medical gloves should be worn when there is an expected risk of visible contamination of the hands with bodily secretions, excreta, or blood.*

**Recommendation** **degree:** ↑↑

**Strength of consensus:** >95%

Pathogen-free disposable medical gloves primarily serve the purpose of interrupting the transmission of nosocomial infections [257], [258] [259], [260]. They are part of personal protective equipment (PPE) and also contribute to occupational safety. They are particularly indicated when the expected pathogens are resistant to ASBHR, such as *C. difficile*, or when the pathogens are especially dangerous, such as viral hemorrhagic-fever causative agents. The need to wear pathogen-free disposable medical gloves in cases of expected high contamination can be justified by the fact that even after hand antisepsis following high contamination, such as contact with bodily secretions, 2 to 3 lg cfu of *Escherichia coli* and methicillin-resistant *Staphylococcus aureus* (MRSA) may still remain on the hands [62], [261]. This is because hand antisepsis on average does not inactivate more than 4 lg cfu [103].

**35. Recommendation**

*Pathogen-free disposable medical gloves should be worn when a risk of transmission of bacterial spores, helminths, protozoa, and oocysts exists. After removing the gloves and performing hand antisepsis, hands should be thoroughly washed.*

**Recommendation** **degree:** ↑↑

**Strength of consensus:** >95%

The necessity arises from the limited efficacy of ABHR against these pathogens [87]. After removing the gloves, it is essential to immediately perform a hygienic hand antisepsis to prevent any potentially adhering bacteria, especially Gram-negative bacteria, from entering and multiplying in the drain. Water for hand washing should only be added after the designated contact time for hand antisepsis has elapsed to avoid prematurely interrupting the antisepsis process.

**36. Recommendation**

*Pathogen-free medical gloves should be changed after providing care to a patient.*

**Recommendation** **degree:** ↑

**Strength of consensus:** >95%

The need to change disposable gloves arises from several factors, including the risk of undetected perforations and high microbial contamination, as hand antisepsis typically inactivates no more than 4 lg units on average. Glove changes are also necessary after contamination with pathogens resistant to ABHR, after engaging in activities that strain the gloves, and after contamination with non-enveloped viruses.

Even though ABHR labeled as effective against non-enveloped viruses are available, they are not recommended for glove antisepsis. This is because ABHR effective against non-enveloped viruses can be harsh on the skin and their compatibility with glove materials is uncertain.

**37. Recommendation**

*Nitrile gloves worn for patient bathing and, if applicable, for changing dressings should be changed after each patient.*

**Recommendation** **degree:** ↑

**Strength** **of consensus:** >95%

The use of nitrile gloves led to a significant increase in the perforation rate after both patient bathing and dressing changes [72].

**38. Recommendation**

*In the following situations, use of hand rub on gloved hands may be considered:*

- *To facilitate the workflow, e.g., consecutive blood drawn from multiple patients, *
- *when changing between unclean and clean activities on the same patient.*

**Recommendation degree:** ↔

**Strength of consensus:** >95%

There are three reasons for the use of rub on gloved hands:

- The efficacy of hand rub on gloved hands is higher than on bare hands and is equivalent to that of the bare hand even when the glove is artificially contaminated with sheep blood [262],
- waste reduction as a contribution to sustainability,
- increasing the awareness of hand antisepsis immediately before aseptic activities: In a stem-cell transplantation unit, hand rub adherence was significantly increased and the number of serious infections tended to decrease by changing gloves only when necessary for the process, and also otherwise disinfecting gloved hands [263].

The following requirements must be considered for ABHRs while wearing gloves:

- The glove must meet the requirements for penetration resistance to microorganisms [264] and be resistant to chemicals [265], [266], [267], [268]. Furthermore, the test of the so-called breakthrough time of 30 min (protection index class 2) must include at least one alcohol.
- The glove has no noted perforations and has not been worn during strenuous activity with increased risk of perforation, e.g., for patient washing or dressing changes [72].
- The glove is not contaminated with blood, secretions, or excretions.
- There is no increased likelihood of contamination with pathogens resistant to alcohol-based hand rubs.

Any information provided by the manufacturer on the number of possible hand rubs on the glove must be followed.

**39. Recommendation**

*Medical gloves should be made of latex-free material.*

**Recommendation degree:** ↑↑

**Strength of consensus:** >95%

Medical disposable gloves do not need to meet the same high requirements for fit and grip as surgical gloves. Therefore, latex-free materials, such as nitrile or vinyl, are recommended due to the risk of developing latex allergies [269]. Since both nitrile and vinyl gloves are not biodegradable, it is worth waiting to see if new biodegradable glove materials are developed, similar to the addition of polysaccharides to natural rubber [270], which would then be preferable.

**40. Recommendation**

*If pathogen-free medical gloves are not provided using an automatic glove dispenser or from a cardboard box that releases the subsequent glove sufficiently when the first one is removed, the retrieval should be done with hands that underwent antisepsis immediately beforehand to avoid touching the box and other gloves.*

**Recommendation degree:** ↑

**Strength of consensus:** >95%

Since conventional glove boxes often result in additional gloves slipping out during retrieval, which can then become contaminated during the process of pushing them back [271], ABHR should be carried out immediately before glove retrieval. With more advanced boxes that have a downward-facing dispensing opening, the risk of such contamination is eliminated [272].

**41. Recommendation**

*Sterile surgical gloves should be worn during all invasive interventions that require barrier measures beyond the basic hygiene procedures, and also when handling sterile medical devices or sterile material. If a sterile gown is worn, the gloves should be pulled over the cuffs.*

**Recommendation degree:** ↑↑

**Strength of consensus: **>95%

This is mandatory [273] because surgical hand antisepsis removes only about 2.0–2.4 lg of the skin flora, but the resident skin flora contains more than 5 lg [48], [119], [120], [274] . The tight closure of the sleeve cuff of the gown is necessary, because after antisepsis of the forearm, 2–3 lg of the resident skin flora remains [137].

**42. Recommendation**

*For sterile surgical gloves, low-latex-allergen surgical gloves with a latex protein content <30 µg/g glove ma* *te* *r* *ial* *should be used.*

**Recommendation degree: **↑↑

**Strength of consensus:** >95%

Concerning medical devices, the focus of allergen exposure is on the use of medical surgical gloves [269], [275], [276]. Because of the allergy risk [277], [278], [279], since 1998 only low-latex-allergen surgical gloves with a latex protein content <30 µg/g glove material must be used in Germany. This is because latex allergies of the immediate type (type I) to natural latex proteins and of the late type (type IV) to auxiliary materials added for production purposes, such as thiuram, represent a considerable problem [280]. Latex allergies can manifest clinically as localized or generalized contact urticaria, bronchial asthma, rhinoconjunctivitis, orolaryngeal and gastrointestinal symptoms, up to and including anaphylactic shock reactions [275], [279], [281], [282]. It is not possible to completely avoid surgical gloves containing natural latex because the comfort, fit and grip have yet to be achieved by any other material [283], [284]. For example, due to the elasticity of latex gloves when performing standardized punctures, the bacterial passage was 10 times lower compared to nitrile and neoprene gloves, so that a higher protective effect can be expected [285]. However, when testing surface mechanical resistance, latex-free neoprene and nitrile gloves were comparable to latex and are an alternative to latex for allergic patients and healthcare workers. In contrast, isoprene was found to be inferior to latex and other non-latex materials [284]. Another latex-free alternative is synthetic rubber, which, however, does not reach the quality of natural rubber.

Due to the use of low-latex or latex-free medical products, the incidence of allergies among nursing staff in Japan in 2014 had decreased significantly compared to 1999 [286].

**43. Recommendation**

*In case of suspected or confirmed latex allergy of both patients and staff, latex-free surgical gloves should be used.*

**Recommendation degree: **↑↑

**Strength of consensus: **>95%

Otherwise, an allergic reaction may be triggered [269], [287], [288], [289].

**44. Recommendation**

*Natural latex-free materials, e.g., made of neoprene or nitrile, should be used for operations on patients with an increased risk of developing a latex allergy (atopy, repeated pediatric surgical operations).*

**Recommendation degree:** ↑

**Strength of consensus:** >95%

Patients suffering from spina bifida, urogenital malformations, esophageal atresia, myelomeningocele and other malformations in need repeated surgeries, as well as patients with pre-existing skin conditions, such as hand eczema and contact urticaria, have an increased risk of developing a latex allergy [290], [291], [292], [293], [294], [295], [296]. Latex-free surgical gloves, latex-free anesthesia, and latex-allergen avoidance in everyday life have significantly reduced latex sensitization prevalence [278], [287].

**45. Recommendation**

*Powdered surgical gloves should not be used.*

**Recommendation degree:** ↑↑

**Strength of consensus: **>95%

Powdered latex gloves have been banned in Germany since 1998 because of the risk of allergy [297]. By using non-powdered gloves, the allergy rate decreased significantly [286].

**46. Recommendation**

*Talc should not be applied to the hands before putting on surgical gloves.*

**Recommendation degree:** ↑

**Strength of consensus: **>95%

Talc and substitute products carry the risk of granuloma formation in the surgical site [298], [299], [300], [301] and are therefore no longer recommended. This risk has not been investigated for an emulsion containing cornstarch; however, since no effect on the amount of sweat was demonstrated [302], its use in surgical gloves is dispensable.

**47. Recommendation**

*In case of increased risk of perforation, such as operations in trauma surgery and orthopaedics (sharp-edged instruments and implants), two pairs of surgical gloves pulled over each other (double gloving) should be worn.*

**Recommendation degree:** ↑

**Strength of consensus:** >95%

Double gloving [303], [304] reduces the risk of injury and thus contamination in case of glove defects, but does not completely prevent it [305], [306], [283]. In two studies, double indicator gloves were compared with double standard gloves. The number of perforations of the inner glove during surgery was not significantly lower when indicator gloves were worn compared with standard gloves [283]. 

**48. Recommendation**

*When only one pair of gloves is worn in visceral surgery, the surgeon and 1* *^st^* *assistant can consider changing gloves after 90 min at the latest, and the 2* *^nd^* *assistant and the operating room nurses can also do so after 150 min at the latest.*

**Recommendation degree:** ↔

**Strength of consensus:** >95%

For the surgeon and the 1^st^ assistant after 90 min at the latest, and for the 2^nd^ assistant and the OR nurses after 150 min at the latest, there was a significant increase in the rate of perforation of surgical gloves with pathogen transfer through the microperforations [305]. 

**49. Recommendation**

*Wearing gloves with an antibacterial barrier or antibacterial impregnation should be avoided.*

Recommendation degree: ↑

**Strength of consensus:** >95%

Gloves with antibacterial barrier or antibacterial impregnation reduce the transfer of pathogens through perforations or the amount of pathogens on the hand [307], [308], [285], but antibacterial impregnation carries a risk of allergy. Since no effect on the SSI rate has been demonstrated to date, their use is not recommended. 

**50. Recommendation**

*If the glove has to be changed due to an intraoperative glove perforation, antisepsis of the affected skin area should be performed. If double gloves are used, it is sufficient to change the defective glove.*

**Recommendation** **degree:** ↑↑

**Strength of** **consensus:** >95%

Hand antisepsis is intended to eliminate pathogens that have reached the hand from the surgical field through the perforation and to prevent them from entering aseptic areas in the event of a renewed microperforation in the further course of the operation. 

If the hand is contaminated with blood or if glove juice has accumulated, the hand must be cleaned with a sterile cloth before antisepsis. 

If the perforation occurred shortly before the end of surgery, it is sufficient to pull a new sterile glove over the perforated glove.

**51. Recommendation**

*If sterile implants (e.g., joint or vascular prostheses) are insert* *ed intraoperatively, gloves should be changed beforehand.*

**Recommendation** **degree:** ↑↑

**Strength of consensus:** >95%

Concerning surgical gloves that were primarily sterile at the start of the operation, by the time of implantation of a sterile hip endoprosthesis, up to 54% of them were contaminated [309]. The contamination rate was even higher before implantation of sterile vascular grafts, at 56–91% for the 1^st^ surgeon, 75–91% for the 1^st^ assistant, and 11–80% for the aseptic instrumentation nurse [310]. The reason for glove contamination is that the deep resident skin flora is only incompletely eliminated by the preoperative skin antisepsis and is released into the surgical field during the course of the operation [311].

**52. Recommendation**

*When wearing air-impermeable medical gloves, because of the perspiration generated, wearing of textile undergloves may be considered.*

**Recommendation** **degree:** ↔

**Strength of** **consensus:** >95%

These are thin cotton gloves for single use or reprocessing. They can be worn under both nonsterile and sterile disposable medical gloves. In both cases, moisture accumulation is counteracted. Their use is considered useful according to TRBA 250 [297] when air-impermeable protective gloves are worn for prolonged periods to reduce hand perspiration. Their use is particularly recommended if irritant dermatosis develops as a result of perspiration. The underglove is changed together with the surgical glove. 

Their use has also been shown to be feasible with non-sterile disposable medical gloves, with a subjectively favorable influence on skin condition due to absorption of moisture, so that nurses and physiotherapists have responded predominantly positively to routine use in patient care [312]. Here, too, the underglove must be changed together with the disposable medical glove

**53. Recommendation**

*Before contact with chemicals – including surface and instrument disinfectants and cytostatics – gloves declared as personal protective equipment (PPE) should be put on.*

**Recommendation degree:** ↑↑

**Strength of consensus:** >95%

If a protective function against chemicals is to be achieved, gloves for high-risk situations (Category III of Regulation (EU) 2016/425 on personal protective equipment, recognizable by the CE marking followed by a four-digit number) should be selected [313]. This quality is recommended for all gloves used in the healthcare sector. Not only the expected protective performance, but above all the assured quality (AQL) is crucial for the protective effect. PPE gloves must meet both the general requirements of DIN EN ISO 21420 [265], especially harmlessness, mechanical strength, ergonomics and resistance to water penetration, as well as the specific requirements related to the intended use, i.e., protection against chemicals and microorganisms, according to EN 374 [264], [266], [267], [268]. For disinfection work, only gloves with a declared protective effect against chemicals and microorganisms guarantee adequate protection. Class 4 gloves with a breakthrough of class 4 should be selected, based on DIN EN ISO 374-4 [268], unless the manufacturer of the surface or instrument disinfectant specifies otherwise.

**54. Recommendation**

*For protection against blood-borne pathogens, especially against viruses, gloves declared as PPE should be worn.*

**Recommendation degree:** ↑

**Strength of consensus:** >95%

The American Standard Test Methods (ASTM) F1671-07 provides information on resistance to pathogens, e.g., viruses [314] transmitted via blood. The examination of the barrier function for cytostatics is currently only regulated by ASTM D 6978-05 [315]. The ASTM can only provide guidance for Europe; however, it is occasionally applied for products on the German market.

**55. Recommendation**

*Household gloves with a long cuff should be used personalized for maintenance cleaning.*

**Recommendation degree:** ↑↑

**Strength of consensus: **>95%

The requirements for household gloves are derived from analogies to protective gloves. Personalization and long cuffs are self-explanatory. At the end of every work shift, the gloves must be discarded or reprocessed. In this case, a validated washing and disinfection process is to be used and hygienic storage is to be assured.

**56. Recommendation**

*When disinfecting patient rooms without known risk of contamination with problematic pathogens, e.g., multi-resistant bacteria or Clostridioides difficile, the household gloves should be disinfected or changed every time the room is changed.*

**Recommendation degree:** ↑↑

**Strength of consensus:** >95%

Because in these cases no contamination with critical pathogens is to be expected, the gloves worn must be disinfected with an ABHR. This will simultaneously contribute to timesavings and to sustainability.

**57. Recommendation**

*Leaving isolation units after disinfecting cleaning of the units, cleaning staff should deposit household gloves for reprocessing or discard them. Subsequently, hand antisepsis should be performed.*

**Recommendation degree:** ↑↑

**Strength of consensus: **>95%

Since household gloves are not as flexible as medical gloves and do not fit the hand well, in case of potential contamination with critical pathogens through disinfection, the glove surface cannot be safely wetted, so that disinfection success is not guaranteed.

### 7. Requirements for hand hygiene

**58. Recommendation**

*In all fields where hand antisepsis for the HCWs is necessary, wearing rings, bracelets, wristwatches or piercings (for example dermal anchor) on hands and forearms (exceptional dosimeters for staff protection) is not allowed.*

**Recommendation degree:** ↑↑

**Strength of consensus:** >95%

Jewellery, including wedding rings, on hands and forearms impede proper hand hygiene, can become a reservoir of pathogens [316], [317], [318], [319] and are therefore not permitted [297]. Furthermore, wearing wedding rings increases the perforation rate of surgical gloves [319]. Rings are also not permissible because of the risk of injury [297].

Ring dosimeters can be worn if they are properly prepared. After wearing ring dosimeters, it is sufficient to soak them in an ABHR for 10 min for disinfection. Thereafter, the ring can be put on the hand again after hand antisepsis and air drying without rinsing with water [320].

**59. Recommendation**

*Fingernails should be cut short and rounded on the fingertips.*

**Recommendation degree:** ↑↑

**Strength of consensus:** >95%

The preconditions for effective hand antisepsis have only been partially investigated, are mainly derived from the hygienic risk assessment, and thus fulfill the criteria of good hospital hygiene (best practice). Short fingernails ending at the fingertips ensure cleaning of the subungual spaces and minimize the risk of glove perforation at the fingertips.

**60. Recommendation**

*It is not allowed to use synthetic fingernails or those treated with gel.*

**Recommendation** **degree:** ↑↑

**Strength of consensus:** >95%

Artificial nails encourage neglect of hand hygiene, increase the risk of perforation of disposable medical gloves and become bacterially colonized with increasing duration of wear, especially at the glued margin, so that no antiseptic effect can be achieved on the nails [321], [322], [323]. Repeatedly, artificial nails have been identified as source of nosocomial infections in immunocompromised patients and of outbreaks of SSIs [324], [325], [326], [327], [328], [329], [330], [331], [332].

**61. Recommendation**

*It is not allowed to use nail polish on fingernails (exception: medical nail polish).*

**Recommendation degree: **↑↑

**Strength of consensus:** >95%

Nail varnish prevents the visual assessment of the nails. With increasing wearing time, colonization on the nails increases and, depending on the age of the varnish, no antiseptic effect is achieved on the varnish [333]. If the indication for the use of bitter-tasting medical nail varnish against nail biting is given, it must be reapplied daily to ensure the effect of hand antisepsis on the nail as well [333]. The team should be informed about the exception.

**62. Recommendation**

*Inflammatory skin lesions on the hands should be covered with a pathogen- and, if necessary, liquid-tight (liquid-tight film) cover after performing a hand rub.*

**Recommendation degree:** ↑

**Strength of consensus:** >95%

Since skin lesions are frequently colonized by microbes [334], [335], covering small lesions or wounds helps prevent the spread of wound infection pathogens.

**63. Recommendation**

*In the case of inflammatory non-purulent skin lesions on the hand, considering the risk, a surgical procedure may be considered, if a sterile covering (film dressing) is applied after surgical hand rub and before donning double surgical gloves.*

**Recommendation degree: ↔**

**Strength of consensus**: >75–95%

These barriers or measures prevent pathogen transfer into the surgical area even in the event of intraoperative microperforation of the outer surgical glove. It may be advisable to consult the occupational health physician.

**64. Recommendation**

*In the case of skin diseases and occupational dermatoses, a consultation with the company’s/institution’s occupational health should be arranged for clar* *i* *fi-cation of causes, therapy planning, and initiation of preventive measures.*

**Recommendation degree: ↑↑**

**Strength of consensus**: >95%

Healthcare workers with acute or chronic skin diseases should present themselves early to the company's/institution’s occupational health service and be informed about the risk of microbial colonization [320], [336], [337]. During the consultation, it is assessed whether colonization with potentially pathogenic organisms is present. Simultaneously, a treatment strategy with the goal of normalizing the skin and its flora [320], [337] is initiated, followed by a skin-care program [337], [338], [339]. The development of protective measures to prevent pathogen transfer from identified carriers to patients should be coordinated with the hygiene team [340]. Risk assessment is necessary. For example, hands colonized with *Serratia marcescens* in a patient with psoriasis caused a nosocomial outbreak [341]. Severe onycholysis and onychomycosis of the right fingernail with concurrent subungual detection of *Pseudomonas aeruginosa* also caused an outbreak, even when latex surgical gloves were worn [342]. Carriers of *S. aureus*, including MRSA, and streptococci were sources of nosocomial infections, with colonized skin lesions preceding nasal colonization with staphylococci as the dominant source [343]. 

**65. Recommendation**

*Wearing short-sleeved work clothing can be considered.*

**Recommendation degree: ↔**

**Strength of consensus: **>95%

If long-sleeved isolation gowns are not required, short-sleeved work clothing has the following advantages. Unlike long-sleeved gowns, there is no risk of contamination at the sleeve end at the wrist. With short-sleeved gowns, the forearm can be included in hand antisepsis if necessary.

Short-sleeved work clothing was introduced due to an outbreak of central venous catheter (CVC)-associated infections. Changing from long-sleeved to short-sleeved work clothing improved hand hygiene compliance to the baseline level. At the same time, the infections stopped. Nurses preferred short-sleeved work clothing after the change [344].

**66. Recommendation**

*Handwashing stations should be located in rooms or in the vicinity of rooms in which medical or nursing pro* *ce* *dures* *are performed, or which serve to prepare such measures, as well as in hospital kitchens, unclean work areas and staff toilets.*

**Recommendation degree: ↑↑**

**Strength of consensus:** >95%

Generally, building codes require a hand wash station in areas in which hand washing is required [83]. Patients’ wash basins can in certain situations be used by the staff, provided that they are equipped with dispensers for ABHR, liquid soap and disposable paper towels. This applies to hand washing after removing gloves after caring for patients with, e.g., *C. difficile*-associated diarrhea or after unexpected massive soiling/contamination of hands in the course of patient care.

**67. Recommendation**

*The hand washing station should be equipped with a sufficiently large, deep sink without overflow with running hot and cold water, wall-mounted, separate dispensers for ABHR, lotion and, if possible, also for skin protection products (alternatively provided in tubes), with disposable hand towels and a waste basket for used paper towels.*

**Recommendation degree: ↑↑**

**Strength of consensus:** >95%

According to the Technical Rules for Biological Agents (TRBA 250), hand washing stations are required to be equipped with a wash basin with hot and cold water, disposable towels with a collection container for discarding used towels, and a wall-mounted dispenser for ABHR [297]. Because of the risk of microbial contamination during use, dispensers are also recommended for providing wash lotion [345] and skin protectant [346], [347], [348]. Skin protection agents can alternatively be provided in tubes.

**68. Recommendation**

*Towel dispensers should be designed to allow easy withdrawal of a towel without contaminating subsequent towels or the opening from which they are removed.*

**Recommendation degree: ↑↑**

**Strength of consensus:** >95%

This requirement is based on indirect evidence stemming from studies that demonstrate contamination of glove boxes [271]. Regular emptying of the waste baskets for used towels must be ensured. Alternatively, retractive dispensers with automatic feed of a textile towel, which is released from a roll and rolled up on a second roll after use, can be considered [121].

**69. Recommendation**

*Electric hot-air hand dryers should not be used in medical or nursing areas.*

**Recommendation degree: ↑↑**

**Strength of consensus:** >95%

Towel dispensers should be preferred over hot-air hand dryers for several reasons, including the lower drying efficacy of hot-air hand-dryers, the lack of mechanical removal of residues (soap residues, dander, skin flora residues), the noise pollution of jet-air dryers, the greater user comfort of towels, and – depending on the electric dryer – a higher risk of pathogen spread [121], [349], [350], [351], [352], [353], [354], [355], [356], [357], [358], [359], [360], [361], [362].

**70. Recommendation**

*Faucets and dispensers for ABHR, wash lotion, and skin protectant should be operable without hand contact* **.**

**Recommendation degree: **↑

**Strength of consensus:** >95%

Using operating levers pushed with the elbow instead of touching with the hand reduces the contact area as a transmission possibility [83]. If sensor-operated taps are used instead of lever-operated taps, it must be checked whether the solenoid valve that releases the water flow is located as close as possible to the tap level, so that no water column can remain in the tap in the resting state in which Gram-negative, non-fermenting microorganisms can settle, which has been reported to be the cause of outbreaks [363], [364], [365], [366], [367], [368], [369].

**71. Recommendation**

*The water flow of the water tap should not be directed into the siphon.*

**Recommendation degree: **↑↑

**Strength of consensus:** >95%

The siphon is an open reservoir of pathogens of patients’ flora [151], [152], [370]. When water enters, bacteria are ejected from the wastewater standing in the siphon over a radius of up to 1.50 m. In cases of siphon contamination >10^5^ cfu/ml, the transmission of bacteria to the hands of nursing staff during hand washing has been demonstrated [152]. Siphons colonized with *P. aeruginosa* have been identified as a risk factor for the colonization of patients with problematic pathogens [151], [152], [153], [371]. Outbreaks of *Enterobacter cloacae, P. aeruginosa, Acinetobacter baumannii* and *Serratia* spp. originating from the siphon have been described [372], [373], [374]. After cleaning the siphon 3 times a day and changing the siphon, an outbreak that had existed for 5 years was ended [374].

If siphon plugs are desired, they should be easy to disinfect and not made of rubber or plastic. It is favorable to have a closure that extends far above the siphon opening to shield the aerosol created by the incoming water. Automatic siphon disinfection systems can be advantageous in special units, e.g., for cystic fibrosis patients, intensive-care neonatology and for the prophylaxis of pseudomonas infections [375].

**72. Recommendation**

*Washbasins should not have an overflow.*

**Recommendation degree: **↑

**Strength of consensus: **>95%

The overflow is a reservoir of pathogens and has been identified as the cause of an outbreak of *Serratia liquefaciens* infections [376]. Washbasins with drainage openings located backwards in the wall reduce environmental contamination [370].

**73. Recommendation**

*Dispensers should be available wherever hand antisepsis needs to be performed regularly. Alternatively, mobile dispensers including gown bottles can be used.*

**Recommendation degree: **↑↑

**Strength of consensus:** >95%

Insufficient equipping with disinfectant dispensers has a negative impact on adherence to hand antisepsis [377], [378]. High compliance with hand antisepsis can only be achieved given an optimal supply and location of dispensers for ABHRs [11]. Therefore, the dispensers must be available wherever hand antisepsis has to be performed regularly, e.g., at the bedside in the patient’s room, at the exit of the room, on dressing trolleys or those used for ward rounds, in locks, etc. [83]. Staff should not have to walk extra distances to access hand antiseptic when performing patient care. The minimum supply is one dispenser per patient bed in intensive care and dialysis units, and one dispenser per two patient beds in non-intensive care units and in the sanitary cell [83]. An insufficient number/location of dispensers for ABHR inevitably leads to neglect of hand antisepsis. For this reason, the WHO recommends making hand antiseptic available “at point of care” [10], in addition to the equipment of hand washing stations prescribed by TRBA 250 [297]. If supplying a sufficient number of wall-mounted dispensers is not achievable, mobile dispenser systems including gown bottles should be made available [83].

**74. Recommendation**

*The replenishment of dispensers for ABHR, washing lotion and skin protection products should be ensured with non-refillable containers (single-use containers).*

**Recommendation degree: **↑

**Strength of consensus:** >95%

If this is not guaranteed, the containers must be aseptically reprocessed before refilling. However, there is no manufacturer recommendation for this. 

For reasons of practicability, it should be possible to use non-refillable liquid soap and hand rub containers from different manufacturers (e.g., Euro dispensers) [379], [380].

**75. Recommendation**

*Hand rubs and liquid soaps used in the dispenser should be easily identifiable. The same applies to the fill level.*

**Recommendation degree: **↑↑

**Strength of consensus:** >95%

Both are a prerequisite for the regular use of the dispensers and to prevent misuse.

**76. Recommendation**

*Dispensers for ABHR, liquid soaps and, if present, skin care lotions should be maintained in such a way that microbial contamination of the pump head and outlet is avoided.*

**Recommendation degree: **↑↑

**Strength of consensus**: >95%

As prerequisite for hygienically safe use, the exterior and interior parts of rigid dispensers, including the drop noses at the outlet, must be easy to clean and disinfect by wiping (follow the manufacturer’s instructions). The dispensers and all permanent parts must be thermo-mechanically reprocessable at an A_0_ value of at least 60°C (e.g. 80°C/1 min). Dispensers with disposable pump heads, which are discarded with the empty container, are preferable. If the pump heads are used for subsequent containers, detailed reprocessing instructions must be provided by the manufacturer [379]. Because of the lower probability of contamination and transmission, dispensing systems that automatically release the rub or wash lotion are to be preferred [379], [380], [381]. Disposable containers in the form of flexible, transparent bags with release of ABHR, wash lotion and skin protection lotion by means of negative pressure instead of rigid containers with release via a pump head, allow the empty bag to be discarded along with its integrated pump outlet, so that the preparation of the dosing pump is not necessary. At the same time, only the outer surfaces of the dispenser need to be disinfected, as the container is disposed of completely. The life cycle assessment is more favorable than with conventional dispensers [379].

Concerning the risk of microbial colonization, soap dispensers are to be assessed more critically than dispensers for ABHR [382], [383]. In addition, after hand contact with the outlet of the soap dispenser, unlike with dispensers for ABHR, the hands are not necessarily decontaminated, but rinsed. Therefore, the use of disposable pumps on the container, which are discarded with the empty container, is also favorable for soap dispensers.

Reprocessing is usually done manually, but is also possible by machine, albeit at higher cost [384].

There are no data upon which to base recommendations for reprocessing intervals. Operating levers should be wiped daily with disinfectant by the cleaning service. The scope and frequency of internal reprocessing of dispensers should be defined in the in-house hygiene plan. The results of microbiological testing of samples routinely taken from dispensers should be taken into consideration. With hygienically adequate dispensers, there is apparently no risk of contamination by pathogens [385].

Outbreaks with contaminated soaps were always caused by open soap bottles or refilled dispenser systems without prior reprocessing, but not by closed systems [382], [383], [385], [386], [387], [388], [389], [390], [391], [392], [393], [394], [395], [396], [397]. Experiments have shown that washing with contaminated liquid soap increases the number of Gram-negative pathogens on the hands and that further spread appears possible even in community facilities [394]. However, even when dispensers are filled with disposable bottles, contamination of the soap is possible if the dispensers, including the riser tube, are not properly cleaned and disinfected over their entire surface [386], [390], [397].

### 8. Skin protection and skin care

**77. Recommendation**

*If the skin is at risk due to work in a damp environment (so-called wet work), fluid-tight gloves should be worn, in addtion to drawing up specific occupational-medical precautions, operating instructions and a skin protection plan. In the skin protection plan, the products for cleaning, protection and care of the skin should be defined. At the same time, possibilities for reducing exposure to moisture should be verified.*

**Recommendation degree: **↑↑

**Strength of consensus: >95%**

Occupational skin diseases have been at the top of the list of occupational diseases for many years [398]. This is due on the one hand to incorrect methods of hand hygiene, i.e., hands are washed too often instead of using ABHR, and on the other to insufficient use of skin protection and skin care products [399]. Depending on the material selected, surgical gloves and the generally frequent wearing of pathogen-free disposable medical gloves can also trigger irritant dermatosis [400]. For the prevention of occupational dermatoses, provision of PPE by the employer is stipulated by law [401], detailed description in [402]. If the skin is endangered by work in a moist environment – this also includes wearing liquid-tight gloves for >2 hours – the employer must provide PPE, draw up operating instructions and a skin protection plan, as well as information on how to reduce exposure to moisture [403]. The preparations for cleaning, protecting and caring for the skin must be specified in the skin protection plan. In case of incipient skin damage, the occupational health service must be consulted immediately.

**78. Recommendation**

*All staff working in medical and nursing care should protect and care for their hands using a suitable product for their skin type (seborrhoeic or sebostatic) with dermatologically proven efficacy.*

*Skin protection products should be used at the start of work and, if necessary, after longer breaks; skin care products should be applied at the end of the working day.*

**Recommendation degree: ↑**

**Strength of consensus:** >95%

Skin protection and skin care primarily serve to prevent irritant dermatoses [218], [220], [404], [405], [406], [407], [408], [409], [410], [411], [412], but are at the same time a pre-condition for effective hand antisepsis [75], as even small tears or microtrauma can become a pathogen reservoir [402], [413]. Rough, cracked skin favors the development of toxic-irritative skin changes (so-called subtoxic cumulative dermatitis) [75], [413] and colonization with potentially pathogenic agents [414]. If irritant substances repeatedly affect the skin in clinically subthreshold concentrations, the skin's buffering capacity and the barrier function may be impaired. Subsequently, noxious substances can penetrate the skin and trigger an inflammatory reaction that can turn into toxic contact dermatitis. As a result, noxious agents can penetrate the skin and trigger an inflammatory reaction that can turn into toxic contact dermatitis. Repeated contact with the irritant at work can lead to chronic hand eczema. In a moist environment (>2 hours water contact/d, wearing gloves >2 hours, washing hands >20 times/d), intercellular substances, especially the epidermal lipids, are released from the stratum corneum and intercellular gaps develop [336]. If the barrier function of the skin is already impaired, as in the case of of individuals who are atopically predisposed, irritants penetrate the skin more rapidly. To prevent irritant toxic contact dermatitis, skin protection and skin care must be systematic and consistent, and preparations with proven efficacy must be used [339], [415]. Skin protection products are applied before work and, if necessary, additionally after longer breaks [415], [416], [417]. Skin care preparations support the regeneration of the skin [418], [419]. The protective effect of skin protection products has been proven in models testing skin irritation [220], [405], [410], as well as in trainees and in the operating theatre [407]. After 3 years of regular training with practical exercises, the prevalence of irritative skin changes in nursing trainees obtained the same order of magnitude at baseline; however, in the control group without training, the prevalence doubled in three years [420]. After application of skin protection and skin care products (3 times/d) in a surgical team, their skin condition was significantly improved without compromising the efficacy of hand antisepsis or increasing the perforation rate of surgical gloves [421]. As there is evidence that some skin care products may interfere with the efficacy of ABHR, their application – unless their influence on hand antisepsis efficacy has been investigated – is best performed at the end of duty. Another reason for this is that skin care products favor the absorption of any chemical residues that may adhere from the work environment, e.g., quaternary ammonium compounds (QAC) after surface disinfection with QAC-based agents [422]. In terms of the effectiveness of the measures, the regular, frequent and correct application of re-fattening external agents proved to be decisive, moreso than the temporal relation to the exposure to water and ABHR. That is, whether the skin had already been treated with a protective agent prior to exposure or with a care product after exposure did not seem to play as important a role [415]. For the selection of skin protection and skin care products, the proof of efficacy according to the guideline “Occupational skin products” [398], the German Social Accident Insurance’s (DGUV) [417] document “Information 212-017” including the results of studies [418], [419], as well as the safety assessment are prerequisites. The occupational physician (in-house or external assistance) should be involved in the decision-making process.

**79. Recommendation**

*If the hands are visibly soiled, the skin should be cleaned before applying skin protection or skin care products.*

**Recommendation degree: ↑**

**Strength of consensus:** >95%

Otherwise, toxic residues from the hospital environment (e.g., traces of surface-active surface disinfectants [422]) embedded in dirt may become fixed on the skin and cause skin irritation with repeated exposure.

**80. Recommendation**

*Products for skin protection and skin care should not contain allergenic additives.*

**Recommendation degree: **↑↑

**Strength of consensus:** >95%

Due to the risk of sensitization, products free of perfumes and preservatives should be selected because repeated application may cause sensitization [398], [423]. 

**81. Recommendation**

*As far as possible, skin protection products without urea or with a urea content of <3% should be used.*

**Recommendation degree: **↑

**Strength of consensus:** >95%

Penetration of chemical residues on the skin (such as traces of desinfectants and medications) is promoted by urea. Thus, and in contrast to cosmetic skin care products, any selected products should contain less than 3% urea [415].

**82. Recommendation**

*Products based on triacylglycerides (natural fatty acids) could be preferable to those based on mineral oils.*

**Recommendation degree: **↔

**Strength of consensus: **>95%

Regarding skin tolerance, natural fatty acids (triacylglycerides) are superior to those based on mineral oils. In skin, triacylglycerides are broken down, thereby releasing fatty acids. These can penetrate the skin better and deeper, achieving their effect in deeper skin layers. Their effects are not limited to provision of fat; many fatty acids support antioxidative and anti-inflammatory processes [424]. It has been shown that glyceride stimulates the endogenous release of glycerol in the skin and improves the hydration and barrier function of the skin [220].

**83. Recommendation**

*Skin protection and skin care products should preferably be provided in dispensers, otherwise in tubes, but not in ointment jars/pots. When tubes are used, care should be taken to avoid back-suction of the expressed strip of ointment into the tube.*

**Recommendation degree: **↔

**Strength of consensus:** >95%

Upon using skin protection and skin care products, the risk of microbial contamination must be considered [425], [426], i.e., ointment jars should not be employed and back-suction of the expressed ointment strip into tubes must be avoided. Otherwise, microbial contamination is inevitable.

### 9. Quality management of hand hygiene implementation

**84. Recommendation**

*The in-house hygiene plan should cover the following points and be easily accessible for all personnel (for instance, on the internet): indications and procedures for hand washing and hand antisepsis, reprocessing of dispensers for ABHRs, wash lotions, skin protection products, selection and use of sterile and non-sterile medical single-use gloves and safety gloves, as well as measures regarding skin care and protection.*

**Recommendation degree: **↑↑

**Strength of consensus: **>95%

In accordance with § 36 of the Infection Protection Act and the hygiene regulations of the federal states, in-house infection hygiene plans must be in place in hospitals as well as all surgeries, dental and medical centers for preventive care, rehabilitation, dialysis and other centers/practices carrying out ambulant interventions in which similar procedures as in hospitals are conducted. Due to the high importance of hand hygiene for the control of nosocomial infections and the interruption of nosocomial outbreaks, it is crucial that any such hygiene plan detail all measures to be taken to stop the transmission of infection via hands. This should include details on how to maintain healthy skin.

**85. Recommendation**

*At the start of their employment, new employees should receive instruction on hand hygiene, and the number of hours of instruction should be documented. At least once a year, all employees should – if possible – be given a refresher training session, which includes updates on current findings, regarding the measures of hand hygiene as well as skin protection and skin,care.*

**Recommendation degree: **↑↑

**Strength of consensus:** >95%

The training of new employees on adherence to the indications and regular conduct of hand antisepsis is a precondition for the implementation of the hygiene plan. Because both nursing staff as well as medical practitioners were found to have obvious deficits in knowledge regarding skin protection and skin care [421], [427], the transfer of knowledge on these topics in connection with establishing a plan for skin protection is important and contributes to an improvement of occupationally irritated skin [428], [429], [430].

The interval for the revision of hygiene plans is specified in the state hygiene regulations at least annually. Thus, in case of updates, employees’ knowledge must also be updated, and if possible complemented by renewed training [83].

**86. Recommendation**

*In* *each facility, interventions for improving the adherence to hand hygiene should be implemented, with the focus on multimodal programs with regular evaluation and feedback.*

**Recommendation degree: **↑↑

**Strength of consensus: **>95%

For hand antisepsis to have an impact on the rate of nosocomial infections, a high compliance of hand antisepsis must be achieved [431]. Interventions with the focus on multimodal programs with regular evaluation and subsequent feedback should be implemented in each facility [83], because it significantly improves compliance [432]. Measuring systems are available to choose from, with direct observation of HCWs as the most accurate option, followed by electronic systems [433], [434] and the determination of ABHR consumption [83]. To evaluate the consumption of ABHR, an interdisciplinary discussion in both the hospital’s organizational unit and hygiene commission should be performed to establish appropriate measures for the improvement of the adherence. In facilities in which no interventions were carried out for hand antisepsis, considerable deficits became apparent, with adherence rates between 5–81% (mean ca. 40%) [11], [432], [435], [436]. It was also clear from the data collected as part of the national hand antisepsis campaign in 2014 in 109 German hospitals that, with an adherence of 72% on average before an intervention, there are still considerable deficits in the daily implementation [437], especially since the Hawthorne-effect can achieve more than 200% [438], [439].

The main reasons for inadequate adherence are lack of interventions, human inadequacies (lack of discipline, indifference, anonymous wrongdoing, forgetting), lack of role models of colleagues or superiors, actual or suspected skin intolerance to the preparations used, unclear instructions, lack of behavioral control, inadequate provision with hand rub dispensers, doubts about the value of hand hygiene, the attitude of employees and information deficits in the area of infection detection, but also lack of staff [10], [377], [378], [440], [441], [442], [443], [444], [445], [446]. At the heart of improving adherence is increasing the awareness and responsibility of the employees for the importance of hand antisepsis for patient protection [447]. The WHO initiated national awareness campaigns with the initiative “Clean care is safer care” [56] to enhance compliance with hand hygiene. In the first step, it is important to deal with the reasons for facility-related non-compliance. However, training as a sole intervention measure only has a brief effect [448], [449]. In contrast, multimodal intervention programs with a focus on regular staff training (training programs) for the implementation of SOPs and their audits, awareness of the role-model effect of superiors, measurement of the hand antisepsis consumption including feedback about the results, improvement of the availability of hand rubs, use of reminders and promotional materials, parallel evaluation of the incidence of nosocomial infections and visible support from administrative levels have proven to be sustainable [10], [432], [435], [436], [450], [451], [452], [453], [454], [455], [456], [457], [458], [459], [460], [461], [462], [463], [464], [465], [466]. Correct implementation of hand antisepsis must also be considered during training measures [435]. Employee training on hand hygiene should be performed at regular intervals (at least annually). To support the German “Clean Hands” campaign, the online “Clinical Hand Hygiene” campaign program was inaugurated by the Professional Association of German Surgeons and the German Society for Hospital Hygiene as a test and learning program [467]. Microbiological examinations, e.g., impression cultures from the hands, can be carried out for special epidemiological questions, but are not suitable for routine testing of the effectiveness of hand antisepsis.

**87. Recommendation**

*To improve hand hygiene compliance, the use of electronic reminder systems may be considered.*

**Recommendation degree:** ↔

**Strength of consensus:** >95%

By combining open and covert observation methods, including regular feedback on adherence to hand antisepsis, e.g., on an electronic board, compliance with hand antisepsis indications can be significantly increased [468]. By installing an electronic system in which a portable transponder communicates with a beacon installed above the patient’s bed, entering and leaving the patient’s environment will be detected. In case of failing to perform hand antisepsis, the transponder sends a signal to the wearer. In addition, the number of hand rubs is recorded using a sensor on the ABHR dispenser. With the anonymous system, WHO moments 1, 4 and 5 are recorded. By using such a system, the compliance was significantly increased by 105% (p<0.000) compared to the initial value of 15%, while also maintaining anonymity. When the system was switched off for 3 months, compliance decreased by 64% but remained above the pre-implementation baseline rate. It becomes clear that compliance can only be maintained with continuous use of the signaling system [434].

**88. Recommendation**

*Stationary units should annually report consumption figures, itemized by organizational units as per the HAND-KISS module, in terms of ABHR units per patient day, to both the nursing and medical administration. Stationary units should also annually evaluate the consumption figures and implement the resulting conclusions.*

**Recommendation degree: **↑

**Strength of consensus:** >95%

In Germany the HAND-KISS module serves to record the consumption of ABHR on stationary units and operating areas (such as dialysis, endoscopy), outpatient medicine, as well as in nursing homes. Optionally, data can be collected as part of direct compliance observation and for provision of dispensers for ABHR. Based on reference data of ABHR consumption, deficits can be identified, stratified by intensive care units/non-intensive care units and medical specialities regarding patient days, and measures can be taken to improve compliance. 

**89. Recommendation**

*Stationary units should participate in the German campaign “Clean Hands”.*

**Recommendation degree: **↑

**Strength of consensus: **>95%

The German campaign “Clean Hands” (“Aktion Saubere Hände”), founded on January 1^st^, 2008, is a national initiative to improve compliance with hand antisepsis in German healthcare facilities. As part of the campaign “Clean Hands”, ABHR consumption levels are documented in the HAND-KISS module. A further option for detecting hand antisepsis adherence of medical staff is direct observation. The collected data can be documented in the HAND-KISS module.

A central condition for good hand antisepsis adherence is the availability of ABHRs at the point of care. The campaign “Clean Hands” defines exact, specific requirements. The provision of dispensers for ABHR can be documented by means of a special form.

The available methods are presented on the NRZ-Homepage (https://www.nrz-hygiene.de/das-nrz). 

**90. Recommendation**

*Outpatient facilities and nursing facilities should annually evaluate the consumption of ABHRs.*

**Recommendation degree: **↑

**Strength of consensus**: >95%

The 5 indications of hand antisepsis should be fulfilled in outpatient facilities [10]. In this regard, knowledge of ABHR consumption is also important in these areas to assess the adherence. Investigations among first-aid personnel in New Jersey (USA) revealed major deficits in the provision of ABHR dispensers and in terms of compliance. The latter was 12% for male and 26% for female personnel before patient contact, 1% and 4% after patient contact and 16% and 19% after invasive procedures [469]. In Dutch medical practices, the compliance of doctors, assistants and nurses was 34%, 51% and 16% [470]. In nursing care facilities, adherence before interventions ranged from 6% to 27% [471], [472], [473], [474], [475]. These results underline the necessity of tracking ABHR consumption in outpatient facilities and nursing-care facilities. The number of daily patient cases serves as an appropriate reference point for orientation. In cases of low consumption, it is advisable to monitor adherence with the 5 WHO indications in practice to facilitate targeted intervention. 

**91. Recommendation**

*In case of an increase in nosocomial infections or amplified spread of multi-resistant pathogens, a bundle of measures should include direct observation of adherence to hand antisepsis.*

**Recommendation degree: **↑ 

**Strength of consensus: >95%**

Because intensified hand antisepsis as part of bundles of measures restricts the spread of multi-resistant pathogens , [17], [19], [21], [22], [23], [24], [476], [477], [478], [479] and controls outbreaks [25], [26], [27], [28], [29], observing adherence can identify behavioral deficits and facilitate targeted interventions. 

### 10. Legal aspects

**92. Recommendation**

*If dispenser containers are refilled with ABHRs that have a marketing authorization as a medicinal product, this should be done in a pharmacy under cleanroom conditions.*

**Recommendation degree: **↑↑

**Strength of consensus: >95%**

Hand rubs with marketing authorization as medicinal product have grandfather status in Germany despite their European classification as biocidal products. According to the irrefutable presumption of Section 2 (4) sentence 1 of the German Pharmaceuticals Act (*Arzneimittelgesetz*, AMG) [480], a product that is approved as a medicinal product under the German AMG is considered a medicinal product. A product that materially does not (or does no longer) fall under the definition of a medicinal product in Section 2 AMG, but has a marketing authorization for a medicinal product, is thus also classified as a medicinal product. In this case, a medicinal product is fictitiously assumed. The drug fictitiousness also applies if the product later no longer meets the corresponding material criteria for classification as a drug. It always applies only to the specific product for which a marketing authorization was granted and only as long as the medicinal product is approved. The drug fictitiousness does not apply to substance-matched products that do not have a marketing authorization. When decanting, the specifications of the AMG must be observed for hand antiseptics that are approved as medicinal products [481], [482]. Section 4 (14) of the AMG defines the decanting of medicinal products, e.g., from larger containers into smaller containers for dispensers, as manufacturing. The person performing the decanting becomes the manufacturer and requires a manufacturing authorization according to Section 13 AMG [483], [481]. Pharmacies and hospital pharmacies fulfill the requirements for decanting as part of their pharmacy operating permit. Only if pharmacies or hospital pharmacies exceed the scope of their pharmacy operating license, must a manufacturing authorization be available. The so-called physician’s privilege under Section 13 (2b) of the German Medicines Act (AMG), i.e., the manufacture of medicinal products without a license, cannot be considered in the area of ABHRs that are approved as medicinal products. This is because the prerequisite would be that the physician manufactures the hand antiseptic for the purpose of personal use on a specific patient. This is logically excluded in the case of hand antiseptics. The legal opinion expressed in section 2.2 of the recommendation by the Commission of Hospital Hygiene and Infection Prevention at the Robert Koch Institute Berlin (KRINKO) entitled “Hand hygiene in health care facilities”, that the decanting of ABHRs with marketing authorization as a medicinal product in doctors’ practices and hospitals is not subject to the requirement of a manufacturing license because they are not professionally manufactured, is legally erroneous and therefore wrong. An activity is considered professional if it is performed on the basis of a profession, in particular a liberal profession, if it is intended to be permanent and if it is for profit. The term “professionally” was included in the wording of the law to supplement the term “commercially” to cover all manufacturing activities carried out for remuneration [484]. Whether or not the activity plays only a minor role is irrelevant to the question of professionalism, because otherwise there would be no need for the privilege of physicians in the manufacture of medicinal products, for example.

**93. Recommendation**

*When decanting ABHRs approved as biocides, the decanting employee should follow all due diligence procedures to ensure safety.*

**Recommendation degree: **↑↑

**Strength of consensus: >95%**

Based on an implementing decision of the European Commission [480] applicable throughout the EU and the EEA, products containing 2-propanol and intended for hand antisepsis, including surgical hand antisepsis, have been considered biocidal products since 2016. The justification for the implementing decision can be transferred in terms of content to other active substances, such as 1-propanol or ethanol. Therefore, a classification as a biocidal product must also be assumed in this respect in the future.

The obligation to obtain a manufacturing authorization does not apply to the decanting of biocidal products. In the case of biocidal products, the person decanting the product is equally obliged under liability law aspects to comply with all duties of care, i.e., for example, all measures to ensure safety. These include cleaning, disinfection and, if necessary, sterilization (in the case of products for surgical hand antisepsis) of the containers before decanting, decanting under aseptic conditions (if necessary, sterile workbench in the case of products for surgical hand antisepsis), documentation of the batch number or decanting date, and implementation by trained personnel [481]. The need for this procedure can be derived from findings on contamination of used solutions. Thus, 1.8% of collected samples (n=16,142) were contaminated, including 70% ethanol. Only PVP-iodine and iodine tincture were not contaminated in any case, which can be attributed to their sporocidal activity. Contamination affected only regional hospitals, and in no case university hospitals [485]. The following risk factors for contamination were identified: Manufacture by untrained personnel, unsuitable containers, and prolonged use. After gas-gangrene infections occurred, the cause was found to be gas-gangrene spores in ethanol used for antiseptic purposes. As a result, spore elimination in ethanol was introduced as a standard formulation (SR) with hydrogen peroxide added, because sterile filtration technology was not available in the former German Democratic Republic (GDR) [486]. For the same reason, the WHO recommends the addition of 1.25% hydrogen peroxide for local production of ABHRs in developing countries [56]. After modification of the original formulations by increasing the alcohol content and reducing the glycerol content, the WHO formulations meet the European normative regulations on efficacy [487]. Since the decanting of ABHRs is not regulated under biocidal product law and thus falls solely within the organizational responsibility of the healthcare facility, either the requirements of pharmaceutical law should be met voluntarily or no decanting should be performed.

**94. Recommendation**

On dispensers or dispenser bottles for hand antiseptics, the contents should be easily recognizable by permanently legible labeling.

**Recommendation degree: **↑↑

**Strength of consensus: >95%**

From a toxicological point of view, misuse of ABHRs dispensers in the patient’s room, provided that they contain only alcoholic active ingredients without addition of remanently active microbicidal agents such as chlorhexidine digluconate, quaternary ammonium compounds or iodophores, is not expected to result in any lasting, serious side effects, as the erroneous oral ingestion of toxicologically critical quantities is not to be expected in cognitively fully responsive patients. In cognitively confused patients, however, access to bottles or dispensers must be prevented, as severe intoxications may otherwise occur [488], [489], [490].

For liability reasons, permanently legible labeling of the dispensers or dispenser bottles with a warning is recommended. This could read, for example: “Alcohol based hand rub for hand use only! Do not drink, splash in eyes or apply to mucous membranes. Fire hazard”. Additionally, pictograms can be attached as a warning [491].

In the case of ABHRs that fall under pharmaceutical law, the labeling requirements under pharmaceutical law must also be observed.

**95. Recommendation**

*The KRINKO recommendation “Hand hygiene in healthcare facilities” should be used as the basis for establishing and implementing hand hygiene measures. Regarding the sanitary-technical requirements for the implementation of hand hygiene measures, the KRINKO recommen* *dation* *“Hygiene requirements for wastewater-carrying systems in medical facilities” should be observed.*

**Recommendation degree: **↑↑

**Strength of consensus: >95%**

According to Section 23 (3) of the German Infection Protection Act (*Infektionsschutzgesetz*, IfSG) [229], managers of hospitals, doctor’s offices, outpatient surgery facilities, day clinics, maternity facilities, emergency services and other facilities mentioned therein must ensure that the measures required according to the state of medical science are taken to prevent nosocomial infections and the further spread of pathogens, especially those with resistance. Compliance with the state of medical science in this area is presumed if the published recommendations of the KRINKO has been observed in each case. This legal presumption not only has consequences under administrative law, e.g., in the case of inspections by the health authorities, but also influences assessments under medical liability law by determining the standard of care. According to Section 630h (1) of the German Civil Code [492], an error on the part of the treating party is presumed if a general treatment risk has materialized that was fully controllable for the treating party and that resulted in injury to the life, body or health of the patient. A fully controllable risk is one that can be excluded with certainty. It is irrelevant to what extent the risk was actually specifically avoidable. Rather, the decisive factor is the assignment of the risk to the sphere of control and organization of the person providing treatment (examples of claims for damages based on errors in hand hygiene in [493]). In contrast, if the source of infection is unclear, a reversal of the burden of proof according to the principles of fully controllable risk is not possible. However, there is a secondary burden of proof on the hospital operator or the physician in the case of alleged hygiene violations. As a rule, the assertion of a hygiene violation is sufficient for this purpose. It is not a precondition for triggering the secondary burden of proof that the patient provide concrete evidence of a hygiene violation. In this case, the treating party has the secondary burden of proof that it has taken the hygiene measures required by the state of medical science. Within the framework of the legal presumption of Section 23 (3) IfSG, this also includes the published recommendations of the KRINKO. Theoretically, the person providing treatment is free to demonstrate and prove that he or she has complied with the relevant standard of care, even without observing the KRINKO recommendations. However, he or she would then have to clearly prove that and how he or she complied with the protection aim of Section 23 (3) IfSG in another way. This proof can be extremely difficult in individual cases. In this respect, the patient's primary burden of proof is limited – to the detriment of the person providing treatment. A special case of treatment error is gross malpractice [examples in [494]. This is medical misconduct that no longer appears comprehensible from an objective medical point of view, because such an error must not be made by the treating physician under any circumstances. The decisive factor is whether the medical malpractice clearly violates established and proven medical knowledge and experience. In accordance with Section 630h (5) of the German Civil Code [492], gross malpractice leads to a reversal of the burden of proof in favor of the patient with regard to the causality of error and damage. Classification as gross malpractice is a legal assessment that is the responsibility of the judge and not the expert. Although an assessment as gross malpractice must find its factual basis in the expert’s explanations, the judge may not leave the assessment to the forensic expert.

## Notes

### Competing interests

The authors declare that they have no competing interests.

### Acknowledgement 

We would like to thank Simone Witzel (Dipl.-Biol.) and Monika Nothacker (MD, MPH), representatives of the Association of the Scientific Medical Societies in Germany, Institute for Medical Knowledge Management (AWMF-IMWi), for their support in the conflict of interest assessment and the formal consensus finding process.

### Funding 

The elaboration of the guideline was not financially supported. 

### Authorization by the participating societies 

The version of the guideline approved by the editorial committee was sent to the boards of the participating societies mentioned above before publication and was authorized and approved by all of them in toto without changes.

### Period of validity and updating procedure 

The guideline was last edited for content on 02/2023, and the validity period is set at 5 years. Comments on the update are welcome.

Contact person is Prof. em. Dr. med. habil. Axel Kramer (axel.kramer@med.uni-greifswald.de).

### Author’s ORCID

Kramer A: 0000-0003-4193-2149

## Supplementary Material

Guideline Report
