# Supplementary material for: S2k-Guideline hand antisepsis and hand hygiene
Source: GMS Hyg Infect Control. 2024 Sep 6;19:Doc42. doi: 10.3205/dgkh000497 (PMC11465089; doi:10.3205/dgkh000497)
Supplement: Guideline Report [file HIC-19-42-s-001.pdf]

Guideline Report

What is new?

New findings on the selection of hand antiseptics and their application, including hygienic requirements and measures to protect the hands, were discussed, possibilities for improving adherence to hand antisepsis were identified, the use of medical gloves and protective gloves as well as requirements for sanitary-technical requirements were specified, and aspects of liability law were added. At the same time, the guideline considers the extension of indications for hand antisepsis under epidemic and pandemic conditions.

In detail, the following aspects were added:

- Possibility of performing hygienic hand antisepsis with shortened exposure time,
- Evaluation of user acceptance before introducing a new hand antiseptic,
- Justification of the harmlessness of ethanol in hand rubs,
- Requirements for disinfection of the gloved hand,
- Influence of short-sleeved professional clothing on the prevention of nosocomial infections,
- Pre-operative elimination of soiling from fingernails, hands and forearms with explanation of the procedure,
- Extension of the range of indications for surgical hand antisepsis,
- Action in case of intraoperative perforation of the surgical glove,
- Requirements for washing lotions and dispensers for liquid soap, alcohol-based hand rubs and skin lotions,
- Requirements for refilling of hand antiseptics,
- Criteria for the use of skin protection and skin care products,
- Causes of insufficient adherence to hand hygiene and intervention strategies for improvement,
- Consequences of the future classification of hand antiseptics as biocides in Europe,
- Liability aspects in the case of nosocomial infections resulting from deficiencies in hand hygiene.

Scope and purpose

The guideline addresses the prevention of nosocomial infections through hygienic and surgical hand antisepsis including hand washing, the use of medical gloves and protective gloves, and provides information on the protection of the community against infection by hand antisepsis. For application in medical and nursing care, the preconditions for ensuring the effectiveness of the measures including skin protection and skin care, measures for increasing adherennce and aspects of liability law are covered.

- Rationale for the selection of the guideline topic
- Hand hygiene is the measure with the highest potential to prevent nosocomial infections
- Target orientation of the guideline
- Transparency of the currently available evidence
- Target patient group
- Patients in inpatient and outpatient care, in epidemic and pandemic situations also the population outside health care facilities
- User target group/addressees

The guideline is aimed at (specialist) physicians/target groups of societies/organisations that were involved in the preparation of the guideline and also patients and people with disabilities and chronic illnesses and serves as information for emergency services, nursing and medical staff working in care facilities, facility management staff working in patient areas, pharmacists, optometrists, tattooists.

Attachment to: Kramer A, Seifert J, Abele-Horn M, Arvand M, Biever P, Blacky A, Buerke M, et al. S2k-Guideline hand antisepsis and hand hygiene. GMS Hyg Infect Control. 2024;19:Doc42. DOI: 10.3205/dgkh000497

Guideline Report

Editorial committee and consensus group

Authors of the guideline

- Prof. Dr. med. em. Axel Kramer (Coordinator)
- Prof. Dr. med. Julia Seifert (Coordinator)
- Bernd Gruber (Coordinator)
- Prof. Dr. med. Marianne Abele-Horn
- Prof. Dr. med. Mardjan Arwand
- Dr. med. Paul Biever
- Dr. med. Alexander Blacky
- Dr. med. Folke Brinkmann
- Prof. Dr. med. Michael Buerke
- Prof. Dr. med. Sandra Ciesek
- Prof. Dr. med. Iris Chaberny
- Prof. Dr. med. Maria Deja
- Prof. Dr. med. Steffen Engelhart
- Dr. med. Dieter Eschberger
- PD Dr. med. Achim Hedtmann
- PD Dr. Dr. med. Julia Heider
- Prof. Dr. med. Udo B. Hoyme
- Dr. med. Christian Jäkel
- Dr. med. Peter Kalbe
- Prof. Dr. med. Horst Luckhaupt
- Prof. Dr. med. Alexander Novotny
- Dr. med. Cihan Papan
- Prof. Dr. med. Hansjürgen Piechota
- Prof. Dr. med. Frank-Albert Pitten
- Veronika Reinecke
- PD Dr. med. Dieter Schilling
- Prof. Dr. med. Walter Schulz-Schaeffer
- PD Dr. med. Ulrich Sunderdiek

Lead professional society:

German Society of Hospital Hygiene; Mandate holder Prof. em. Dr. med. habil. Axel Kramer (Representative: Prof. Dr. med. Frank-Albert Pitten) (2023)

Participating professional societies (and approval date of the guideline by the respective professional society):

- Paul-Ehrlich-Society for Infection Therapy; mandate holder Prof. Dr. med. Marianne Abele-Horn (2023)
- German Society for Oral, Maxillofacial and Facial Surgery; Mandatsträgerin PD Dr. Dr. med. Julia Heider (2023)
- German Society for Virology; mandate holder Prof. Dr. med. Sandra Ciesek (2023)
- German Society for Hygiene and Microbiology; mandate holder Prof. Dr. med. Iris Chaberny (2023)
- Society of Hygiene, Environmental and Public Health Sciences; mandate holder Prof. Dr. med. Steffen Engelhart (2023)
- German Society for Orthopaedics and Orthopaedic Surgery; mandate holder PD Dr. Achim Hedtmann (2023)
- Professional association of specialists in orthopedics and trauma surgery; mandate holder PD Dr. Achim Hedtmann (2023)

- German Society for Gynecology and Obstetrics; mandate holder Univ.-Prof. Dr.med. Prof. Dr. h.c. Udo B. Hoyme (2023)
- German Cardiac Society; mandate holder Prof. Dr. Michael Buerke (2023)
- German Society for Hospital Hygiene; mandate holder Prof. Dr. med. em. Axel Kramer (representative: PD. Dr. med. Frank-Albert Pitten) (2023)
- German Society for Surgery; mandate holder Prof. Dr. med Alexander Novotny (2023)
- German Society for Urology; mandate holder Prof. Dr. med. Hansjürgen Piechota (2023)
- German Society for Digestive and Metabolic Diseases; mandate holder PD Dr. med. Dieter Schilling (2023)
- German Society for Trauma Surgery; mandate holder Prof. Dr. med. Julia Seifert (2023)
- German Society of Pediatrics and Adolescent Medicine; mandate holder Dr. med. Cihan Papan (2023)
- German X-ray Society; mandate holder PD Dr. Ulrich Sunderdiek (2023)
- German Society of Anaesthesiology and Intensive Care Medicine; mandate holder Prof. Dr. med. Maria Deja (2023)
- Professional Association of German Surgery; mandate holder Dr. med. Peter Kalbe (2023)
- German Society for Pediatric Infectious Diseases; mandate holder Dr. med. Folke Brinkmann (Vertreter Prof. Dr. med. Arne Simon)
- German Society for Internal Medicine; mandate holder Dr. med. Ay Mehtap (2023)
- German Nursing Council; mandate holder Bernd Gruber (2023)
- German Society for Internal Intensive Care and Emergency Medicine; mandate holder Dr. med. Paul Biever (2023)
- Federal Working Group for Self-Help of People with Disabilities and Chronic Illness and their Family Members; mandate holder Andrea Trenner (2023)
- Austrian Society for Hospital Hygiene; mandate holder Dr. med. Alexander Blacky (2023)
- General Accident Insurance Institution Austria; mandate holder Dr. Dieter Eschberger (2023)
- German-speaking interest group of infection prevention experts and hospital hygiene consultants; mandate holder Veronika Reinecke (2023)

Other participants in the editorial committee (without voting rights in the consensus process):

- Prof. Dr. med. Mardjan Arvand (Head of FG 14 at the Robert Koch Institute)
- Dr. Christian Jäkel (Medical Law)

Guideline Report

Guideline development stage

S2k

Guideline development process

This is a second fundamental update of an S2k guideline (first version dated 09/21/2016), which means a consensus-based guideline with a representative panel as well as structured consensus finding.

The guideline was developed by the Working Group Hospital and Practice Hygiene of the Association of Scientific Medical Societies (AWMF), an interdisciplinary group of experts, representative of the topic and the target group. The participating professional societies were involved in the guideline development.

The method of the S2k guideline (consensus-based + interdisciplinary matching) is based on the recommendations of the Association of Scientific Medical Societies (AWMF). For the formulation of the recommendations, a systematic literature review (until December 2022) with evaluation of the sources took place in the interdisciplinary dialogue of the participating mandate holders of the professional societies and associations.

The recommendations were graduated according to the following three-level scheme:

**Description**	**Expression mode**	**Symbol**
Strong recommendation	shall/not shall	⇈ / ⇊
Recommendation	should/ not shoud	↑ / ↓
Recommendation open	can be considered/waived	↔

Membership of the guideline group, participation of stakeholders

The societies mentioned under “Editorial Board and Consensus Group” were involved in the development and formal consensus of the guideline. In addition, a draft version of the updated guideline was made available to all professional societies of the AWMF for critical review and comment. By involving the broadest possible expertise, the guideline aimed to achieve the highest possible validity.

Search, selection and evaluation of scientific evidence

The literature search considered the recommendation “Hand Hygiene in Healthcare Facilities” of the Commission of Hospital Hygiene and Infection Prevention at the Robert Koch-Institute Berlin (KRINKO) and references relevant to the guideline development (original papers, reviews, meta-analyses).

Development of the guideline

The editorial committee prepared the draft of the updated guideline on the basis of the literature and coordinated it with all mandate holders in attendance. Subsequently, the draft of the guideline was made available to the members of the consensus group (all mandate holders) for critical review and comment within the framework of a Delphi process with written independent expert consultation. The comments of the members of the consensus group were integrated into a new version, submitted to the members of the editorial/consensus group for another critical review and finalized at the consensus conference.

Declaration of interests and handling of conflicts of interest

All participants in the guideline submitted their declarations of interest (AWMF table for declarations of interest in guideline projects) to the coordinator. In the table form, the participants were asked to indicate whether and, if so, which thematic reference to the guideline/guideline topic exists for the interests presented.

The following evaluation criteria were used:

- Paid expert/consultant activities for industrial companies;
- Participation in a scientific advisory/advisory board: paid activity for industrial companies;
- Lectures: paid by industry;
- Authorship or co-authorship: only if industry controlled;
- Research projects/conduction of clinical studies: directly or partly financed by industrial companies;
- Ownership interests (patents, shareholdings) with guideline relevance;
- Indirect interests with relevance.

On the basis of the table, possible conflicts of interest were discussed, and management decisions were made. For this purpose, the information was reviewed regarding an existing thematic reference, thematic relevance, type and intensity of the relationship. In the sense of active conflict of interest management, it should be decided, depending on the content of the declarations of interest of the members of the editorial committee, whether the vote of a mandate holder should not be included in the consensus determination procedure for individual recommendations due to possible conflicts of interest. Possible conflicts of interest were classified as low/moderate/high. Low conflicts of interest due to fees for lectures (financed by relevant industry) lead to a limitation of the AG management function, but have no consequences for the vote. Possible moderate conflicts of interest were seen especially in the case of memberships in advisory boards and lecturing activities with fees from the industry, if the content was about hand hygiene issues (consequence: no vote). A high conflict of interest would have existed in the case of holding patents in connection with hand hygiene or activities predominantly for the industry (consequence: no vote and no discussion on the topic).

The relevance of activities or interests for a possible conflict of interest was assessed individually. The assessment and unanimous confirmation were made by the editorial committee at its meeting on February 3, 2023. The handling of interests was subsequently assessed and confirmed again jointly by all participants at the consensus conference, also on February 3, 2023. Resulting, a moderate conflict of interest was identified in four cases. Insofar as recommendations were voted on for which the moderate conflict of interest was relevant, these four mandate holders were not considered in the consensus determination procedure.

Consensus finding

For consensus finding, the finalized guideline with all recommendations was provided by the editorial committee. The formal consensus process was conducted for all guideline recommendations at the consensus conference on 2/3/2023.

The following steps were followed in the consensus conference: NIH (National Institutes of Health)-type consensus conference (the recommendations were voted under neutral moderation by Dipl.-Biol. Simone Witzel and Dr. med. Monika Nothacker, MPH as follows): Presentation of the recommendations to be voted in plenary by the working group, opportunity for questions and submission of justified amendments, voting on the recommendations and amendments. If necessary: discussion, development of alternative proposals and final vote with following consens grading:

Strong consens	consent of >95% of the participants
Consens	consent of >75–95% of the participants
Majority consent	consent of >50–75% of the participants
No majority consent	consent of <50% of the participants

At the consensus conference on Feb. 3, 2023 (28 societies represented), approval was granted by the mandate holders for all recommendations as reflected in Chapter 1 of the guideline for all societies represented. With one exception (“consensus”), all recommendations were approved with “strong consensus”.
